# Supplementary material for: Numerical assessment of wake-based estimation of instantaneous lift in flapping flight of large birds
Source: PLoS One. 2023 May 4;18(5):e0284714. doi: 10.1371/journal.pone.0284714 (PMC10159204; doi:10.1371/journal.pone.0284714)
Supplement: S1 Appendix — (PDF) [file pone.0284714.s001.pdf]

Aerodynamic polar used in the simulations

The polar used in the simulations is the one of an AS6092 profile. It is part of a series of bird-like airfoils designed by Ananda and Selig [1]. The polar and the profile are represented in Fig 1. As the airfoil is cambered, the lift coefficient at zero angle of attack is positive, and it has a negative pitching moment.

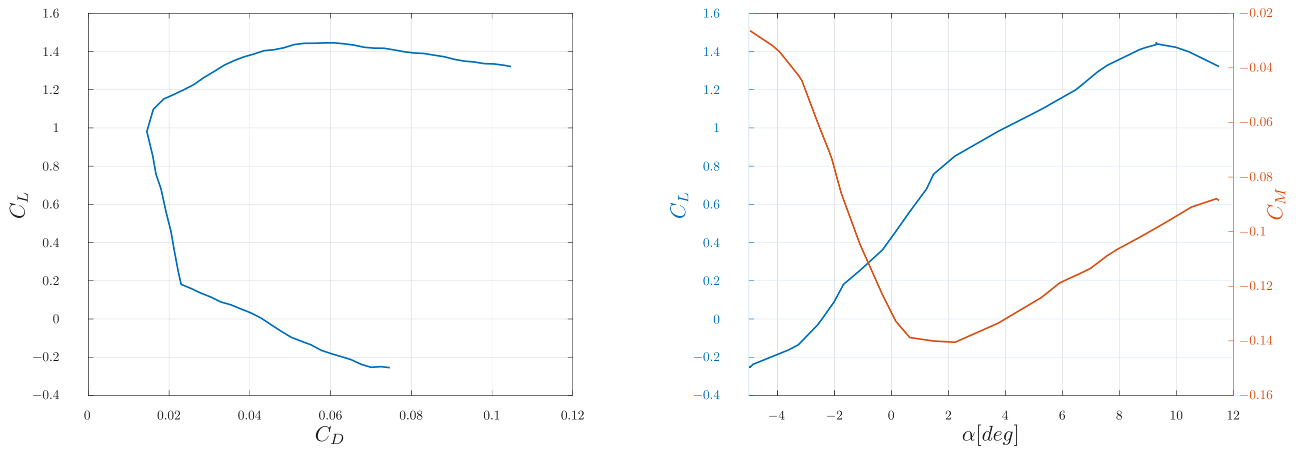

(a) Polar

(b) Lift and Moment coefficients

Figure 1: Polar used in the simulations. (a): lift and drag coefficients. (b) lift and moment coefficient as a function of the angle of attack.

During the simulations, the values of C_L, C_D and C_M are obtained through an interpolation between the values found in the polar. When the angle of attack of a profile exceeds the range of values represented in Fig 1, the first or last values of the coefficients are considered, meaning the the polar is extended with a flat line. As the angle of attack does not often exit the range of the data, we consider this assumption to be acceptable.

References

[1] Gavin K. Ananda and Michael S. Selig. Design of bird-like airfoils. In *2018 AIAA Aerospace Sciences Meeting*, page 0310, 2018.
