# Supplementary material for: Numerical assessment of wake-based estimation of instantaneous lift in flapping flight of large birds
Source: PLoS One. 2023 May 4;18(5):e0284714. doi: 10.1371/journal.pone.0284714 (PMC10159204; doi:10.1371/journal.pone.0284714)
Supplement: S2 Appendix — (PDF) [file pone.0284714.s002.pdf]

Derivation of the lift estimation formula from the momentum flux

Between Eqs (3) and (4) of section 1.2, we transform the momentum flux term into a more compact form. We thus start from

$$\frac{\mathbf{F}_{momflux}}{\rho} = -\oint \hat{\mathbf{n}} \cdot \mathbf{u}\mathbf{u}\,\mathrm{d}S\ . \tag{1}$$

When considering only the lift component of Eq. (1), we get

$$-\left(-\hat{\mathbf{e}}z\right) \cdot \oint \hat{\mathbf{n}} \cdot \mathbf{u}\mathbf{u}\,\mathrm{d}S = \oint \hat{\mathbf{n}} \cdot \mathbf{u}\, u_z\,\mathrm{d}S\ . \tag{2}$$

If the closed surface used for the integration of Eq. (2) extends to infinity in the y and z directions and towards the inflow, the contributions of these surface will drop to zero as we discussed in section 1.2. The only remaining integral is thus the one on the plane normal to x in the outflow, where the normal is $\hat{\mathbf{n}} = -\hat{\mathbf{e}}_x$. We thus have

$$\oint \hat{\mathbf{n}} \cdot \mathbf{u}\, u_z\,\mathrm{d}S = -\int_{S_{out}} u_x u_z\,\mathrm{d}S\ . \tag{3}$$

Equation (3) can be separated into two components:

$$-\int_{S_{out}} u_x u_z\,\mathrm{d}S = U_\infty \int_{S_{out}} u_z\,\mathrm{d}S - \int_{S_{out}} \left(U_\infty + u_x\right) u_z\,\mathrm{d}S\ . \tag{4}$$

The first integral of Eq. (4) can be integrated by parts:

$$\int_{S_{out}} u_z\,\mathrm{d}S = \int_{S_{out}} \left(\frac{\partial}{\partial y}\left(y\, u_z\right) - y\frac{\partial u_z}{\partial y}\right)\mathrm{d}S\ . \tag{5}$$

The first term of Eq. (5) corresponds to the variation of $y\, u_z$ between points infinitely far in the positive and negative y directions. As the velocity due to the wake vortices decay with $1/r^2$, this converges to zero as the outflow plane extends. The second term of the integral can be transformed to make the streamwise component of the vorticity appear ($\omega_x = \frac{\partial u_z}{\partial y} - \frac{\partial u_y}{\partial z}$)

$$-\int_{S_{out}} y\frac{\partial u_z}{\partial y} = -\int_{S_{out}} \left(y\,\omega_x + y\frac{\partial u_y}{\partial z}\right)\mathrm{d}S \tag{6}$$

The second term of Eq. (6) can be split into separate integrals along y and z

$$\int_{S_{out}} y\frac{\partial u_y}{\partial z}\,\mathrm{d}S = \int_{-\infty}^{\infty} y\left(\int_{-\infty}^{\infty} \frac{\partial u_y}{\partial z}\,\mathrm{d}z\right)\mathrm{d}y = 0\ , \tag{7}$$

which tends to zero as the outflow plane extends to infinity because the integral in the z direction is null.

Thus, we finally obtain

$$\int_{S_{out}} u_z\,\mathrm{d}S = -\int_{S_{out}} y\omega_x\,\mathrm{d}S\ . \tag{8}$$
