# Supplementary material for: Numerical assessment of wake-based estimation of instantaneous lift in flapping flight of large birds
Source: PLoS One. 2023 May 4;18(5):e0284714. doi: 10.1371/journal.pone.0284714 (PMC10159204; doi:10.1371/journal.pone.0284714)
Supplement: S3 Appendix — (PDF) [file pone.0284714.s003.pdf]

Ratio of the circulatory to added mass lift forces for a 2D wing in plunging motion

Consider a plunging wing in 2D with a lift coefficient following

$$C_l = 2\pi\alpha \ ,$$

with α being the instantaneous angle of attack, and an added mass of

$$m_a = \rho\,\pi\,c^2/4 \ ,$$

with c being the wing chord. The vertical position of the wing results from a harmonic motion

$$z(t) = A\sin\left(\omega\,t\right) \ ,$$

with A being an amplitude and $\omega = 2\pi\,f$ the angular frequency of the plunging motion.

The instantaneous angle of attack and acceleration of the wing are respectively given by

$$\alpha = \mathrm{atan}\left(-\frac{\dot{z}}{U}\right) = \mathrm{atan}\left(-\frac{\omega\,A\cos\left(\omega\,t\right)}{U}\right) \ ,$$

and

$$\ddot{z} = -\omega^2 A\sin\left(\omega\,t\right) \ .$$

The vertical forces due to the effect of the angle of attack and to the added mass are thus expressed as

$$C_{l,\alpha} = 2\pi\,\mathrm{atan}\left(-\frac{\omega\,A\cos\left(\omega\,t\right)}{U}\right) \ ,$$

and

$$C_{l,am} = \frac{2m_a\,\ddot{z}}{\rho\,U^2\,c} = \frac{\pi\,c}{2U^2}\left(\omega\right)^2 A\sin\left(\omega\,t\right) \ .$$

If the vertical velocity of the wing is small compared to the horizontal velocity U the atan can be simplified to its argument. In this case, the ratio of the amplitudes of both forces contributions is expressed as

$$\frac{C_{l,\alpha}}{C_{l,am}} = \frac{4\pi\,U\,\omega\,A}{\pi\,c\,\omega^2\,A} = \frac{4U}{\omega\,c} = \frac{2}{k} \ .$$

where $k = \frac{\omega\,c}{2U}$ is called the reduced frequency of the motion.

We can thus see that the ratio between these two contributions to the vertical force of a purely plunging airfoil is inversely related to the reduced frequency, and that the larger its value, the more important the added mass effects.
