# Supplementary material for: Numerical assessment of wake-based estimation of instantaneous lift in flapping flight of large birds
Source: PLoS One. 2023 May 4;18(5):e0284714. doi: 10.1371/journal.pone.0284714 (PMC10159204; doi:10.1371/journal.pone.0284714)
Supplement: Attachment — Submitted filename: Paper_Review.pdf [file pone.0284714.s005.pdf]

Paper Review

Manuscript ID: PONE-D-22-25640
Title: Numerical assessment of wake-based estimation of instantaneous lift in flapping flight

This paper presents a numerical investigation of a flapping bird to assess the validity of various wake-based theories of instantaneous lift estimation, which are widely used experimentally. The flapping flight of a large bird in a Reynolds number of 10^5 is modeled numerically using a vortex particle-mesh-based fluid solver. The authors have shown that the circulatory instantaneous lift component of a constant velocity flapping bird can be approximated with good accuracy from a single cross-flow plane behind it (assuming viscous effects are neglected), while the added-mass lift component cannot be retrieved. If the flapping bird is known to accelerate/decelerate during the flight, it is best that the instantaneous lift estimation would be performed as closest as possible to the bird.

Generally, the paper is well-written, well-organized, and presents great insights and significance to the field of flapping bird aerodynamics. However, the current manuscript version still requires some revisions before being accepted. Here are the three main comments I have:

1. The numerical setup utilized in the current paper is not detailed fully, omitting large portions that are relevant for the reader to follow. Here are a few parts that need to be adequately described in the paper: bird modeling, discretization, computational domain, numerical schemes, fluid/motion coupling, etc.
2. The current version of the paper ignores a substantial portion of existing literature in the context of instantaneous lift estimation from the near-wake measurements behind flapping birds relevant to the present study. Here are a few examples that computed the unsteady drag and lift forces of freely flying birds, worth mentioning in the paper:
   [1] Ben-Gida H., Kirchhefer A. J., Taylor Z. J., Bezner-Kerr W., Guglielmo C. G., Kopp G. A., and Gurka R. (2013) "Estimation of unsteady aerodynamics in the wake of a freely flying European starling (Sturnus vulgaris)", *PLOS ONE* 8(11), p. e80086. DOI: https://doi.org/10.1371/journal.pone.0080086
   [2] Stalnov O., Ben-Gida H., Kirchhefer A. J., Guglielmo C. G., Kopp G. A., Liberzon A., and Gurka R. (2015) "On the estimation of time dependent lift of a European starling (Sturnus vulgaris) during flapping flight", *PLOS ONE* 10(9), p. e0134582. DOI: https://doi.org/10.1371/journal.pone.0134582
   [3] Nafi A., Ben-Gida H., Guglielmo C. G., and Gurka R. (2020) "Aerodynamic forces acting on birds during flight: A comparative study of a shorebird, songbird and a strigiform", *Experimental Thermal and Fluid Science* 113, p. 110018. DOI: https://doi.org/10.1016/j.expthermflusci.2019.110018
3. Since the paper analyzes the case of a large flapping bird flying at a relatively high Reynolds number, I suggest the authors change the paper title to: "*Numerical assessment of wake-based estimation of instantaneous lift in flapping flight of large birds*".

Apart from the above main comments, below are more specific comments on the paper. Moreover, the paper consists of some minor grammar mistakes and typos to address, which I listed in the attached .pdf file.

<u>Specific comments for the authors to address</u>:

1. In the Introduction, lines 25-26, pp. 1: "*In order to experiment on a flying bird without hindering it with sensing equipment, one often relies on measurements in the wake of the animal.*" – Please re-write the sentence to clarify your meaning. What does it mean "to experiment on a flying bird"?
2. In the Introduction, lines 32-33, pp. 2: "*Usually, circulation is considered to be zero at the start of the downstroke but this remains an estimation.*" – This statement is somewhat misleading. What circulation do you refer to? The total circulation generated by the bird? Circulation generated by the wings? Circulation generated by the tail? The circulation value could be different depending on the integration area of the vorticity field. Please revise the sentence to be more specific. Also, please present a reference for your statement.
3. In the Introduction, lines 49-51, pp. 2: "*In [11], Wang et al. simulated the flow around and behind flapping wings to evaluate the validity of a lift estimation method and propose a correction of said method that enables an accurate estimation of the average lift*." – Please indicate what lift estimation method was studied by Wang et al. (2019).
4. In the Introduction, lines 54-55, pp. 2: "*In these conditions, the viscous and added mass effects have a lesser impact and we expect the unsteady lift to be recoverable further in the wake*." – Please provide references to support your statement of neglecting the added mass for your case of study. Also, please elaborate on why do you expect that the unsteady lift can be recoverable further in the wake"?
5. In the Introduction, lines 58-61, pp. 2: "*We finally assess the validity of the lift estimation techniques in numerical experiments using an in-house model of a flapping bird [12], which is based on a vortex particle-mesh method and faithfully simulates the unsteady wake of the bird over large distances*." – What are numerical experiments? Please use different wording. In addition, please add a reference to the vortex particle-mesh method.
6. The quality of Figures 1 and 3 is very poor. Please improve the quality of these figures to better capture the flow details.
7. In Section 2.1, lines 72-76, pp. 3: "*The whole bird is represented as a single line, which is obtained by extracting one line for each wing and merging them together to account for the influence of the body. At each time-step, the wing lines are computed from the position of the bones and feathers embodied into the bio-mechanical model. They are defined as a line passing through the quarter of the chord – i.e. the line linking the leading and trailing edges – normal to it at each point. In the center, the chord length is set to the length of the bird body*." – How did you model and account for the bird's body in your computation? Was it treated as a bluff body? Something else? Moreover, did you model the bird's head or tail? The tail, in particular, can significantly affect the overall lift of the bird [1,2]. If not considered in the computations, the authors would need to present a solid argument as to why they think the tail interaction with the wake is negligible. Generally, I suggest the authors include a more representative three-dimensional image of the modeled bird (instead or in addition to Figure 2), so it would be easier for the reader to follow the actual bird geometry studied here.

[1] Norberg A. R. (1994) "Swallow tail streamer is a mechanical device for self-deflection of tail leading edge, enhancing aerodynamic efficiency and flight manoeuvrability", The Royal Society 257, pp. 227-233.

[2] Usherwood J. R. et al. (2020) "High aerodynamic lift from the tail reduces drag in gliding raptors", The Journal of Experimental Biology 223. DOI: https://doi.org/10.1242/jeb.214809

8. In Section 2.1, lines 79-81, pp. 3: "*In the body region, the aerodynamic performance of the profile are degraded in terms of lift-to-drag ratio. We thus divide the lift coefficient by 3 in the simulations. This captures the higher contribution of the body to the drag than to the lift of the whole bird.*" –What do you mean by "*We thus divide the lift coefficient by 3 in the simulations*"? Please elaborate in the text to better describe your modeling approach of dividing the lift coefficient by 3, as the reasoning is unclear.
9. In Section 2.1, line 82, pp. 3: "*The profile used in these simulations is an AS6092 airfoil [14]. It is a bird-like airfoil and its polar is available in [14], where it has been computed using XFOIL [15].*"
   a. It needs to be clarified why the AS6092 airfoil was used in this study, as opposed to other bird-like airfoils available in Ananda and Selig (2018). The authors should include their reasoning for modeling the bird wings with such an airfoil section in the text.
   b. Birds' wings are complex lifting surfaces that are characterized by sweep, camber distribution, thickness distribution, twist distribution, and chord distribution [1-5]. Did you consider any of the above wing characteristics in the modeling presented in the paper? Please elaborate in the manuscript text to better describe the modeled bird's wings.

      [1] Nachtigall W. and Wieser J. (1966) "Profilmessungen am taubenflúgel", *Zeitschrift fur vergleichende Physiologies* 52, pp. 333-346.
      [2] Oehm H. and Kitzler U. (1975) "On the geometry of the avian wing," Studies on the Biophysics and Physiology of Avian Flight II", NASA TT-F-16901, 1975.
      [3] Withers P. C. (1981) "An aerodynamic analysis of bird wings and fixed aerofoils", *Journal of Experimental Biology* 90, pp. 143-162.
      [4] Liu T., Kuykendoll K., Rhew R., and Jones S. (2006) "Avian wing geometry and kinematics", *AIAA Journal* 44(5), pp. 954-963.
      [5] Klän S., Bachmann T., Klaas M., Wagner H., and Schröder W. (2009) "Experimental analysis of the flow field over a novel owl based airfoil," *Experiments in Fluids* 46(5), pp. 975-989.

10. In Section 2.1, line 87, pp. 3: Please indicate how you calculated the wake lambda's wavelength.
11. Figure 1 – Please indicate in the figure, and also in the text, the size/dimensions of the computational domain (control volume) used to perform the simulations presented in this paper. What are the streamwise/lateral/normal extents?
12. In Section 2.1, line 97, pp. 4: "*The default values of the kinematic parameters are provided in table 1.*" – Please include the reference from which the default kinematics of the bird's wing was evaluated.
13. Figure 2 – It would be helpful if the authors would mark the seven joints on the wing.
14. In Section 2.1, Eq. 2, pp. 5: Please include a reference to the computation of the added mass matrix expressed in equation 2.
15. The authors used somewhat misleading terminology in the paper to describe their two numerical simulations, namely, experiment 1 and experiment 2. I suggest the authors revise the paper text and use different terminology to describe their numerical simulations, thus avoiding confusion. A better terminology could be 'numerical scenarios' (scenario 1 and scenario 2).
16. Figure 4,5,6 – I suggest the authors normalize the results of the normal force. Maybe, using the bird's weight so that the results could be better interpreted. Also, it could be helpful if the authors would mark the downstroke and upstroke phases of the flapping cycle on the figures.
17. In Figure 4 – Please elaborate on why the small amplitude 'spikes' appear along the normal force curves (particularly noticeable for the total force). Also, the legend in Figure 4 is missing.

18. In Section 4, lines 317-319, pp. 12: "*The wake also presents root vortices that have been observed in [18, 6, 7] and spanwise vortices due to time-variations of the wing circulation, as in [4, 9].*" – The bird's wakes in Figure 3 seems to be characterized by 2-4 layers of concentrated spanwise vorticity (with alternating sign) aligned at an angle with respect to the horizon. Gurka et al. (2017) referred to the quadruple layers of concentrated spanwise vorticity as 'double branch' features and noticed they exist in the wake of three different bird species when the circulation gradient changes sign, suggesting a common role for unsteady aerodynamics in lift generation for flapping birds. I recommend that the authors refer to Gurka et al. (2017) in their discussion of the simulated bird wake to further augment the manuscript's findings.
19. In Section 4.2, lines 374-375, pp. 13: "*Although the quality of the estimation remains acceptable at a distance of seven spans, we see that it degrades as the integration plane moves further downstream from the bird*". – Does this mean that the instantaneous lift component can only be recovered using the estimation from **near-wake** flow measurements behind flapping birds? If so, I think this is a solid finding to be included in the paper.
20. In the Conclusions, lines 382-383, pp. 14: "*The simulation framework mimics the conditions of a wind tunnel…*". – This is somewhat inaccurate since you did not model the wind tunnel walls with an impermeable/no-slip boundary condition in your numerical setup. Therefore, I suggest the authors remove their mention of mimicking wind tunnel tests with their numerical simulations from the paper text.
