# Supplementary material for: Numerical assessment of wake-based estimation of instantaneous lift in flapping flight of large birds
Source: PLoS One. 2023 May 4;18(5):e0284714. doi: 10.1371/journal.pone.0284714 (PMC10159204; doi:10.1371/journal.pone.0284714)
Supplement: Attachment — Submitted filename: PONE-D-22-25640_reviewer.pdf [file pone.0284714.s006.pdf]

PLOS ONE

Numerical assessment of wake-based estimation of instantaneous lift in flapping flight

--Manuscript Draft--

**Manuscript Number:**	PONE-D-22-25640
**Article Type:**	Research Article
**Full Title:**	Numerical assessment of wake-based estimation of instantaneous lift in flapping flight
**Short Title:**	Numerical assessment of wake-based estimation of instantaneous lift in flapping flight
**Corresponding Author:**	Victor Colognesi<br>UCLouvain<br>Louvain-la-Neuve, Brabant Wallon BELGIUM
**Keywords:**	bird; bird flight; PID; bird wake; wake; lift; force estimation; simulation; flapping
**Abstract:**	Experimental characterization of bird flight without instrumenting the animal requires measuring the flow behind the bird in a wind tunnel. Models are required to establish the relationship between measured velocities and the corresponding aerodynamic forces. Widely-used models can however prove inconsistent when evaluating the instantaneous lift. Yet, accurately estimating variations of lift is critical in order to reverse-engineer bird flight. In this work, we revisit mathematical models of lift based on the conservation of momentum in a control volume around a bird. Using a numerical framework to represent a flapping bird wing and compute the flow around it, we mimic the conditions of a wind tunnel and produce realistic wakes. Providing ground truth measurements of the flow everywhere around the simulated bird, we assess the validity of several lift estimation techniques. We observe that the circulation-based component of the instantaneous lift can be retrieved from measurements of velocity in a single plane behind a bird, with a delay that is found to depend directly on the free-stream velocity. We further show that the lift contribution of the added-mass effect cannot be retrieved from such measurements, and quantify the level of approximation due to ignoring this contribution to instantaneous lift estimation.
**Order of Authors:**	Victor Colognesi
	Renaud Ronsse
	Philippe Chatelain
**Additional Information:**	
**Question**	**Response**
**Financial Disclosure**<br><br>Enter a financial disclosure statement that describes the sources of funding for the work included in this submission. Review the submission guidelines for detailed requirements. View published research articles from *PLOS ONE* for specific examples.<br><br>This statement is required for submission and **will appear in the published article** if the submission is accepted. Please make sure it is accurate.	Victor Colognesi is supported by a FRIA grant (Grant number FC 21291) from the Fonds de la Recherche Scientifique de Belgique (F.R.S.- FNRS, https://www.frs-fnrs.be/en/).<br><br>This work is part of a project supported by the ARC program (grant number 17/22-080, RevealFlight) of the Federation Wallonie-Bruxelles (https://www.federation-wallonie-bruxelles.be).<br><br>Computational resources have been provided by the Consortium des Equipements de Calcul Intensif (CECI), funded by the Fonds de la Recherche Scientifique de Belgique (F.R.S.-FNRS) under Grant No. 2.5020.11 and by the Walloon Region.<br><br>The present research also benefited from computational resources made available on the Tier-1 supercomputer of the Federation Wallonie-Bruxelles, infrastructure funded by the Walloon Region under the grant agreement 1117545.<br><br>The funders had no role in study design, data collection and analysis, decision to publish, or preparation of the manuscript.

Powered by Editorial Manager® and ProduXion Manager® from Aries Systems Corporation

**Unfunded studies**<br>Enter: *The author(s) received no specific funding for this work.*<br><br>**Funded studies**<br>Enter a statement with the following details:<br>• Initials of the authors who received each award<br>• Grant numbers awarded to each author<br>• The full name of each funder<br>• URL of each funder website<br>• Did the sponsors or funders play any role in the study design, data collection and analysis, decision to publish, or preparation of the manuscript?<br>• **NO** - Include this sentence at the end of your statement: *The funders had no role in study design, data collection and analysis, decision to publish, or preparation of the manuscript.*<br>• **YES** - Specify the role(s) played.<br><br>* typeset	
**Competing Interests**<br><br>Use the instructions below to enter a competing interest statement for this submission. On behalf of all authors, disclose any competing interests that could be perceived to bias this work—acknowledging all financial support and any other relevant financial or non-financial competing interests.<br><br>This statement is required for submission and **will appear in the published article** if the submission is accepted. Please make sure it is accurate and that any funding sources listed in your Funding Information later in the submission form are also declared in your Financial Disclosure statement.<br><br>View published research articles from *PLOS ONE* for specific examples.	The authors have declared that no competing interests exist.

**NO authors have competing interests**<br><br>Enter: *The authors have declared that no competing interests exist.*<br><br>**Authors with competing interests**<br><br>Enter competing interest details beginning with this statement:<br><br>*I have read the journal's policy and the authors of this manuscript have the following competing interests: [insert competing interests here]*<br><br>* typeset	
**Ethics Statement**<br><br>Enter an ethics statement for this submission. This statement is required if the study involved:<br><br>• Human participants<br>• Human specimens or tissue<br>• Vertebrate animals or cephalopods<br>• Vertebrate embryos or tissues<br>• Field research<br><br>Write "N/A" if the submission does not require an ethics statement.<br><br>General guidance is provided below. Consult the submission guidelines for detailed instructions. **Make sure that all information entered here is included in the Methods section of the manuscript.**	N/A

**Format for specific study types**<br><br>**Human Subject Research (involving human participants and/or tissue)**<br>• Give the name of the institutional review board or ethics committee that approved the study<br>• Include the approval number and/or a statement indicating approval of this research<br>• Indicate the form of consent obtained (written/oral) or the reason that consent was not obtained (e.g. the data were analyzed anonymously)<br><br>**Animal Research (involving vertebrate animals, embryos or tissues)**<br>• Provide the name of the Institutional Animal Care and Use Committee (IACUC) or other relevant ethics board that reviewed the study protocol, and indicate whether they approved this research or granted a formal waiver of ethical approval<br>• Include an approval number if one was obtained<br>• If the study involved *non-human primates*, add *additional details* about animal welfare and steps taken to ameliorate suffering<br>• If anesthesia, euthanasia, or any kind of animal sacrifice is part of the study, include briefly which substances and/or methods were applied<br><br>**Field Research**<br><br>Include the following details if this study involves the collection of plant, animal, or other materials from a natural setting:<br>• Field permit number<br>• Name of the institution or relevant body that granted permission	
**Data Availability**<br><br>Authors are required to make all data underlying the findings described fully available, without restriction, and from the time of publication. PLOS allows rare exceptions to address legal and ethical concerns. See the PLOS Data Policy and FAQ for detailed information.	Yes - all data are fully available without restriction

A Data Availability Statement describing where the data can be found is required at submission. Your answers to this question constitute the Data Availability Statement and **will be published in the article**, if accepted.<br><br>**Important:** Stating 'data available on request from the author' is not sufficient. If your data are only available upon request, select 'No' for the first question and explain your exceptional situation in the text box.<br><br>Do the authors confirm that all data underlying the findings described in their manuscript are fully available without restriction?	
**Describe where the data may be found in full sentences. If you are copying our sample text, replace any instances of XXX with the appropriate details.**<br><br>• If the data are **held or will be held in a public repository**, include URLs, accession numbers or DOIs. If this information will only be available after acceptance, indicate this by ticking the box below. For example: *All XXX files are available from the XXX database (accession number(s) XXX, XXX.).*<br>• If the data are all contained **within the manuscript and/or Supporting Information files**, enter the following: *All relevant data are within the manuscript and its Supporting Information files.*<br>• If neither of these applies but you are able to provide **details of access elsewhere**, with or without limitations, please do so. For example:<br><br>*Data cannot be shared publicly because of [XXX]. Data are available from the XXX Institutional Data Access / Ethics Committee (contact via XXX) for researchers who meet the criteria for access to confidential data.*<br><br>*The data underlying the results presented in the study are available from (include the name of the third party*	The 3D velocity and vorticity fields collected in the wake of the bird for experiment 1 are available at https://doi.org/10.14428/DVN/KQQAQJ.<br><br>2D velocity and vorticity fields collected at three downstream distances during experiment 2 are available at https://doi.org/10.14428/DVN/7ATEGZ.

and contact information or URL).<br>• This text is appropriate if the data are owned by a third party and authors do not have permission to share the data.<br><br>* typeset	
Additional data availability information:	

Powered by Editorial Manager® and ProduXion Manager® from Aries Systems Corporation

Manuscript Click here to access/download;Manuscript;manuscript.pdf

Numerical assessment of wake-based estimation of instantaneous lift in flapping flight

Victor Colognesi, Renaud Ronsse, Philippe Chatelain
UCLouvain; Institute of Mechanics, Materials and Civil Engineering
Louvain-la-Neuve, Belgium

September 15, 2022

Abstract

Experimental characterization of bird flight without instrumenting the animal requires measuring the flow behind the bird in a wind tunnel. Models are required to establish the relationship between measured velocities and the corresponding aerodynamic forces. Widely-used models ~~can~~ ~~however~~ prove inconsistent when evaluating the instantaneous lift. Yet, accurately estimating variations of lift is critical in order to reverse-engineer bird flight. In this work, we revisit mathematical models of lift based on the conservation of momentum in a control volume around a bird. Using a numerical framework to represent a flapping bird wing and compute the flow around it, we mimic the conditions of a wind tunnel and produce realistic wakes. Providing ground truth measurements of the flow everywhere around the simulated bird, we assess the validity of several lift estimation techniques. We observe that the circulation-based component of the instantaneous lift can be retrieved from measurements of velocity in a single plane behind a bird, with a delay that is found to depend directly on the free-stream velocity. We further show that the lift contribution of the added-mass effect cannot be retrieved from such measurements~~,~~ and quantify the level of approximation due to ignoring this contribution to instantaneous lift estimation.

1 Introduction

Bird flight has been a source of inspiration for ages. While modern aerodynamics leads to ~~ever~~ better understanding of the mechanisms behind flapping flight, it is still not perfectly understood. The impressive performance of the bar-tailed godwit – which has been found to migrate for 11000 km without stopping or feeding [1, 2] – is but an ~~example~~ among ~~others~~ of the efficiency of bird flight. It is thus of great interest for both biologists and engineers to observe and study birds ~~in order~~ to further understand the complex mechanisms governing their flight.

The study of bird ~~flight~~ ~~however~~ involves many challenges. In order to experiment on a flying bird without hindering it with sensing equipment, one often relies on measurements in the wake of the animal. PIV (Particle

Image Velocimetry) allows such measurements by measuring the velocity of the fluid along a surface in the wake, like in [3]. Models are then required to compute the aerodynamic forces from these velocity signatures in the wake.

These models can be based on various assumptions. In the works of Spedding et al. [4] and Henningsson et al. [5], for example, the spanwise vorticity is evaluated (using PIV in a transversal plane). Since the spanwise vorticity is linked to the temporal variations of the wing circulation, the estimation of this circulation at an instant of the flapping period is required. Usually, circulation is considered to be zero at the start of the downstroke but this remains an estimation.

Other works consider the streamwise vorticity. This component of the vorticity is shed from the wing trailing edge as its circulation varies along the span. Since the circulation reaches zero at the wingtips, any wing producing lift will thus leave a wake with a streamwise component of vorticity. Measuring this component enables other methods for the estimation of the lift, as it is done in the works of Henningsson et al. [6], Muijres et al. [7], KleinHeerenbrink et al. [8] or Johansson et al. [9], among others.

While multiple methods exist to compute the lift of a bird from wake measurements, it has been showed by Gutierrez et al. [10] that commonly used methods do not necessarily agree with each other, and that their results can be dependent on the streamwise distance between the bird and the measurement plane. While most experimental measurements of bird wakes are achieved in wind tunnels where the distance between the bird and the measurement plane is constant, in [10], the bird flies through a fixed measurement plane in still air. This experiment highlights the deformations occurring in the wake and shows that they can affect the output of commonly used lift estimation methods. It also means that the wake of a bird is not necessarily frozen, as it is often assumed.

The results of [10] could be caused by vortex breakdown, as they hypothesize, or by non-zero streamwise velocities, as suggested in [9]. In either case, 3D data is necessary to further understand the evolution of the wake and how to use it to estimate the lift of the bird producing it. Numerical frameworks are useful tools to generate 3D velocity fields in the wake of a bird, which would be difficult to measure experimentally. In [11], Wang et al. simulated the flow around and behind flapping wings to evaluate the validity of a lift estimation method and propose a correction of said method that enables an accurate estimation of the average lift. However, the proposed estimation fails to recover the unsteady lift of the flapping wing.

The present work focuses on the flapping flight of a large migratory bird. This case is characterized by a higher Reynolds number and a lower reduced frequency than in [11]. In these conditions, the viscous and added mass effects have a lesser impact and we expect the unsteady lift to be recoverable further in the wake.

In this paper, we propose a derivation of an expression allowing to estimate the instantaneous lift of a bird in flapping regime from velocities measured in the wake. At every step of the development, we highlight the assumptions that lead to a formula commonly used to compute the lift from such data. We finally assess the validity of the lift estimation techniques in numerical experiments using an in-house model of a flapping bird [12], which is based on a vortex particle-mesh method and faithfully simulates the unsteady wake of the bird over large distances. While the lift of gliding birds and the average lift of flapping birds have been succesfully estimated using similar tools as the ones used in the present paper, existing literature does not report a robust method to evaluate

instantaneous lift ~~in~~ flapping regime.

2 Materials and methods

2.1 *In silico* wind tunnel

The simulation framework used to produce the results is based on the model presented thoroughly in [12]. It consists of a bio-mechanical model of a bird skeleton coupled to a vortex particle-mesh~~-~~based fluid solver. The wing is represented in the latter using an Immersed Lifting and Dragging Line (ILDL)[13]. The whole framework is set to mimic the conditions of an experimental wind tunnel. The simulation domain is thus subjected to a constant inflow at the forward flight velocity of the bird so that it remains steady. These simulated experimental conditions are represented in Fig. 1.

Figure 1: Control volume around the bird and outflow plane in its wake. The tubes behind the bird represent the vorticity in its wake, and the red and blue shapes in the outflow plane represent the vorticity, with the color corresponding to the sign of its streamwise component. The axes of the reference frame used throughout the paper are also represented.

The whole bird is represented as a single line, which is obtained by extracting one line for each wing and merging them together to account for the influence of the body. At each ~~time-step,~~ the wing lines are computed from the position of the bones and feathers embodied into the bio-mechanical model. They are defined as a line passing through the quarter of the chord – i.e. the line linking the leading and trailing edges – normal to it at each point. In the center, the chord length is set to the length of the ~~bird~~ body.

At each section of the wings, the lift and drag are computed based on the local relative velocity, using polar data, ~~i.e.,~~ the values of the lift and drag coefficients of a 2D profile as a function of the angle of attack and the Reynolds number. In the body region, the aerodynamic performance of the profile ~~are~~ degraded in terms ~~of~~ lift-to-drag ratio. We thus divide the lift coefficient by 3 in the simulations. This captures the higher contribution of the body to the drag than to the lift of the whole bird.

The profile used in these simulations is an AS6092 airfoil [14]. It is a bird-like ~~airfoil~~ and its polar is available in [14], where it has been computed using XFOIL [15]. Vortical structures related to the production of lift and drag are shed from the ILDL. Vortex particles are emitted to represent the shedding due to the variations of circulation along the line and through time. In addition, vortex dipoles are shed to represent the signature of drag in the wake. More detail on these methodological aspects can be found in [13].

In the present case, the simulated bird is a *Geronticus Eremita* with a mass of 1.2 kg and flying at a velocity of $15\,\mathrm{m\,s^{-1}}$. Its flapping frequency f is 4 Hz and the wavelength of the wake λ is thus equal to 3.75 m. The simulation domain is subjected to a constant inflow at a velocity of $U_\infty = 15\,\mathrm{m\,s^{-1}}$, ensuring that the bird remains at a fixed

position in the simulation frame. With a mean aerodynamic chord of the wing $\bar{c} = 0.18$ m, we define the Reynolds number $Re = U_\infty \bar{c}/\nu = 1.8\ 10^5$ and reduced frequency $k = \omega\bar{c}/2U_\infty = 0.15$, where ν is the kinematic viscosity of the fluid ($\nu = 1.48\,10^{-5}\mathrm{m}^2\mathrm{s}^{-1}$ for air) and $\omega = 2\pi f$.

The wing kinematics result from the rotations of seven joints in the wing skeleton, represented in Fig. 2. Each joint angle q_i follows a pure sinusoidal trajectory, governed by a unique frequency $f = 4$ Hz, and joint dependent mean value $q_{0,i}$, amplitude A_i and phase ϕ_i. Each joint i thus follows a trajectory defined as $q_i(t) = q_{0,i} + A_i \sin(2\pi f t + \phi_i)$. The parameters $q_{0,i}$, A_i and ϕ_i are adjusted in order to resemble experimentally observed bird wing kinematics. The default values of the kinematic parameters are provided in table 1.

Joint	Shoulder Y	Shoulder X	Shoulder Z	Elbow Z	Wrist Y	Wrist Z	Wrist X
$q_{0,i}$ [deg]	0.63	0	9.59	-16.67	0	16.67	0
A_i [deg]	2.14	22.22	11.11	16.67	1.85	16.67	0
$\phi_{0,i}$ [rad]	$-\pi/2$	π	$-\pi/2$	$\pi/2$	$-\pi/2$	$-\pi/2$	N.A.

Table 1: Kinematic parameters used in the simulations for the right wing (the left wing is symmetric)

Closed loop controllers are used to stabilize and trim the flight. That is, the bird adapts the trajectories of certain joints to ensure that it stays at an equilibrium and remains on average at the same velocity and altitude. Four parameters are selected for this purpose: the mean values of the pitching and sweeping angles of the shoulder joint ($q_{0,s,Y}$ and $q_{0,s,Z}$) and the amplitude of the pitching and flapping motion of the shoulder joint ($A_{s,Y}$ and $A_{s,X}$). The values of these parameters are determined through the use of PID controllers taking as input the period-averaged values of the altitude, forward flight velocity and pitch angle and their reference values. The wing kinematics and the stabilization of the *in silico* bird are presented with more detail in [12].

Figure 2: Representation of the bird skeleton and the joints between the rigid bodies. Both rotations of the wrist, characterized by the angles $q_{w,Z}$ and $q_{w,Y}$ are represented as examples.

The simulation framework does not currently compute the added mass effect on the wing and the related aerodynamic forces. This phenomenon being due to potential effects, it mostly affects the flow directly around the wing, and it does not influence the flow further behind. However, it can influence the evolution of the vortical structures in the wake shortly after their shedding. The added mass contribution to the aerodynamic force can be computed *a posteriori* from the wing kinematics. To evaluate this force, we consider each wing profile to behave like an ellipse with a length equal to the chord c and a width equal to the thickness t of the profile. The acceleration of the profile is obtained from the simulation and the local force is computed as if it was a 2D flow. For the three degrees of freedom of the 2D profile (i.e. the vertical and horizontal translations and the pitching rotation), the added mass forces are expressed as

$$\mathbf{F}_{am} = -\boldsymbol{M}_a \mathbf{a} \ , \qquad (1)$$

where $\mathbf{F}_{am} = [F_z, F_x, M_y]^T$ is the added mass force vector expressed in the local 2D frame, $\mathbf{a} = \left[\ddot{z}, \ddot{x}, \ddot{\theta}\right]^T$ is the 2D acceleration vector and $\boldsymbol{M}_a$ is the added mass matrix

$$\boldsymbol{M}_a = \rho \begin{pmatrix} \frac{\pi\, c^2}{4} & 0 & -\frac{\pi\, c^3}{16} \\ 0 & \frac{\pi\, t^2}{4} & 0 \\ -\frac{\pi\, c^3}{16} & 0 & \left(\frac{c^2}{4} - \frac{t^2}{4}\right)^2 \end{pmatrix} \tag{2}$$

2.2 General expression of the aerodynamic force acting on a flying body

The aerodynamic force acting on a flying object – a bird in this case – results from an exchange of momentum between it and the fluid. The conservation of momentum in a control volume around the bird allows to express the aerodynamic force from the velocity and pressure of the fluid in and around this volume. Indeed, the aerodynamic force $\mathbf{F} = [F_x, F_y, F_z]^{\mathrm{T}}$ acting on an impermeable body in a static volume V bounded by a surface S can be expressed as

$$\frac{\mathbf{F}}{\rho} = -\frac{\mathrm{d}}{\mathrm{d}t}\int_V \mathbf{u}\,\mathrm{d}V + \oint_S \hat{\mathbf{n}} \cdot \left[-\frac{p}{\rho}\boldsymbol{I} - \mathbf{u}\mathbf{u} + \boldsymbol{T}\right]\,\mathrm{d}S\ , \tag{3}$$

where $\mathbf{u} = [u_x, u_y, u_z]$ is the local velocity vector at every point inside of the volume, $\hat{\mathbf{n}}$ the normal to the surface, p the pressure at every point on the surface S, ρ the fluid density, $\boldsymbol{I}$ the unit matrix and $\boldsymbol{T} = \mu\left(\boldsymbol{\nabla}\mathbf{u} + \boldsymbol{\nabla}\mathbf{u}^{\mathrm{T}}\right)$ is the viscous stress tensor, with μ being the dynamic viscosity of the fluid.

The interpretation of Eq. (3) is straightforward. The force resulting from exchanges of momentum with the fluid corresponds to the increase of momentum in such fluid, which is the first integral of Eq. (3). If the volume is finite, however, the momentum of the fluid inside it is affected by fluxes of momentum at its boundaries and by external forces acting on the same boundaries. This has to be corrected when evaluating the aerodynamic force and corresponds to the second integral of Eq. (3).

Although Eq. (3) provides a simple and exact expression of the aerodynamic force, it requires access to the velocity field in an entire volume and to the pressure on a closed surface, both of which can prove difficult in real-life experiments. Instead, it is preferable to rely on integrals of the velocity and its derivatives on surfaces around the bird, and ideally on a single surface behind it called the *outflow* plane. Examples of a control volume and a candidate outflow plane are shown in Fig. 1.

Taking Eq. (3), Noca et al. [16] used vector calculus and the Navier-Stokes equations to obtain expressions of the instantaneous aerodynamic force a flier is experiencing using only integrals of the velocity and its derivatives on surfaces around it, thus getting rid of the pressure in the expression of the force. This resulting "flux equation" of [16] can be re-written as

$$\begin{aligned} \frac{\mathbf{F}}{\rho} \;&=\; \oint_S \hat{\mathbf{n}} \cdot \left(\frac{1}{2}u^2\boldsymbol{I} - \mathbf{u}\mathbf{u}\right)\,\mathrm{d}S + \frac{1}{2}\oint_S \mathbf{x} \times \left(\hat{\mathbf{n}} \times (\mathbf{u} \times \boldsymbol{\omega})\right)\,\mathrm{d}S \\ &-\; \frac{1}{2}\frac{\mathrm{d}}{\mathrm{d}t}\oint_S \hat{\mathbf{n}} \cdot \left[(\mathbf{x}\cdot\mathbf{u})\,\boldsymbol{I} - \mathbf{x}\mathbf{u} + 2\mathbf{u}\mathbf{x}\right]\,\mathrm{d}S\ , \end{aligned} \tag{4}$$

where $\boldsymbol{\omega} = \boldsymbol{\nabla} \times \mathbf{u}$ is the vorticity vector. To derive Eq. (4) from the "flux equation" of [16], it is assumed that the flow is incompressible and that the contribution of the viscous terms to the estimation of aerodynamic forces is

negligible. Since the case that we study is set at a high Reynolds number ($Re = 10^5$) and at a low Mach number, these assumptions are reasonable. The time-dependency of the variables of Eq. (4) are omitted for the sake of clarity. Unless stated otherwise, any field or variable will be considered dependent on time in the rest of this paper.

Equation (4) provides an expression of the aerodynamic force that only depends on the velocities on a closed surface around a flying object. While this is more achievable than volume integrals, it is still challenging to access the velocity all around a bird. In this work, we focus only on the vertical component of the force which, on average, compensates the weight and corresponds to the lift force in level flight. To further simplify the equations and develop an expression of the lift that can be evaluated using experimental measurements, more hypotheses are required. In the following sections, we thus make some further assumptions on the velocity field in the wake of a bird and how they can be used to simplify Eqs (3) and (4) and apply them to the lift estimation.

2.3 Assumptions on the velocity field in a bird wake

The velocity field resulting from the lift forces acting on the bird can be predicted in a simplified way, especially concerning the scaling of the velocity field when moving away from the bird and its wake. The measured velocity is induced by two lift contributions: the wing circulation and the added-mass effect.

The effect of the wing circulation in the wake can be roughly represented by a horseshoe vortex. Reality is more complex, and even if the vorticity takes the shape of a horseshoe, its intensity and width will vary with the position. But this simple representation allows us to build intuition about the trends of the velocity field. This vortex structure induces a flow that is mostly two-dimensional in the wake and with a velocity magnitude decaying with $1/r^2$ if r is the distance in the $y - z$ plane to the center of the wake.

Any integral of the velocity on a closed surface surrounding the bird is divided into three parts: an integral on a plane normal to the free stream velocity in the wake (also called outflow plane), an integral on the surfaces surrounding the wake, parallel to the free stream velocity, and an integral on a plane normal to the free stream velocity upstream from the bird (called inflow plane). The integral on the outflow plane is necessarily non-zero because the vortex pair crosses the surface, inducing non-zero velocities in it. An integral of the velocity on the lateral planes tends to zero as the volume extends in the lateral directions because of the quadratic decay of the velocity. However, an integral of the velocity multiplied by a distance to the vortex wake (y or z) can converge to a non-zero value because the size of the integration surface then also increases with r. An integral of the velocity on the inflow plane also tends to zero as the distance between the plane and the bird increases because of the absence of vorticity upstream from the bird.

The velocity field related to the added mass effect can be modeled by the flow induced by a set of source-sink dipoles situated on the wing. The intensity of these dipoles is related to the time-varying velocity of the wing. The velocity induced by a single source or sink decays with $1/r^2$ but this is changed to $1/r^3$ in the case of a dipole (with r being the distance to the center of the dipole in the 3D space). The same applies to the finite set of dipoles when evaluating the velocity at a large distance much larger than span of the wing.

When integrating the velocity induced by the added mass, there is no reason to separate the outflow plane from the lateral ones. The integral over a closed surface around the bird thus tends to zero as the volume expands unless the velocity is multiplied by the distance to the bird (x, y or z), in which case the integral can converge to a non-zero value.

2.4 Lift estimation from an outflow plane–theory

From the observations made in section 2.3, we can see that the first integral of Eq. (4) is only non-zero on the outflow plane, provided that the control volume extends far enough in the lateral directions. These integrals only show a contribution from the added mass if the plane is very close to the bird. Since the vorticity field is limited to the vortical structures in the wake, the second integral of Eq. (4) can only be non-zero on the outflow plane.

The third integral of Eq. (4) contains contributions from both sources of lift (circulation and added-mass). The contribution of the lateral planes to this integral cannot be neglected because of the multiplication of the velocity $\mathbf{u}$ with the position $\mathbf{x}$.

Comparing Eqs (3) and (4), we retrieve the momentum flux in both equations. The time-derivative integral of Eq. (3) is equivalent to the last term in the last integral of Eq. (4). This means that all the remaining terms in Eq. (4) correspond to the effect of the pressure on the control volume.

An expression of the lift is obtained through the scalar product of the force vector $\mathbf{F}$ by a unit vector in the vertical direction $-\hat{\mathbf{e}}_z$. Since the pressure only contributes to the lift on the horizontal planes of the control volume, it is possible to build a control volume that extends far enough in the vertical direction so that this term tends to zero. In this case, the only remaining terms correspond to the variation of momentum in the volume and the momentum flux at its boundaries.

Since the volume integral of Eq. (3) cannot be computed in experimental setups, the only way to measure its contribution is through its expression in Eq. (4). However, it may also prove difficult to compute this integral on planes all around a bird. This is one of the reasons why most experimental works are based on the momentum flux measured in a single outflow plane, located behind the animal. As stated in section 2.3, the integral for the momentum flux tends to zero for all the other planes, so integrating on a large outflow plane should accurately represent the actual value.

Estimating the lift based on the momentum flux in the wake implies the assumption that the momentum travels in the wake at a steady speed. A measurement at a time t at a distance x behind a bird flying at a velocity U_∞ then corresponds to the lift of the bird at a time $t - x/U_\infty$. However, for this to be true, all the momentum injected into the fluid at that precise time has to exit through the plane at the same moment. This would not be the case if either the distance over which the momentum has to be transported varies (if the wing has a significant amount of sweep) or if the speed at which the momentum is transported varies in the wake. Note that this latter condition corresponds to the frozen-wake hypothesis.

After developments reported in appendix A, the momentum flux term can be expressed as

$$\frac{L}{\rho} = \frac{\mathbf{F} \cdot (-\hat{\mathbf{e}}_z)}{\rho} = -U_\infty \int_{S_{out}} y\, \omega_x \, \mathrm{d}S - \int_{S_{out}} (u_x + U_\infty)\, u_z \, \mathrm{d}S \ , \tag{5}$$

where U_∞ is the magnitude of the inflow velocity vector (aligned with $-\hat{\mathbf{e}}_x$). Contrarily to what the signs might indicate, the expression $u_x + U_\infty$ represents departures from the free stream velocity.

The first term of Eq. (5) corresponds to a classical fixed-wing formula, that can also be derived from lifting line theory and is usually associated with the Kutta-Joukowsky theorem. It will be called the *KJ* term. The second term results from the deficit or surplus of the streamwise component of the velocity and its effect on the transport of momentum, involving the difference $u_x + U_\infty$. It will be called the *velocity departure* or *VD* term.

The developments leading to Eq. (5) require the outflow plane to extend to infinity. This means that the lateral and horizontal planes of the control volume are positioned infinitely far from the bird. The contribution of these planes to the integral of the pressure and to the momentum flux, therefore becomes negligible. As seen before, though, the integral corresponding to the time-derivative does not tend to zero as these planes go to infinity.

While an infinite outflow plane is required to evaluate all the velocity in it, it is not the case for the vorticity, which forms a compact field. Since vorticity is contained in a region that does not extend far in the y and z directions, any plane encompassing this region will correctly evaluate the *KJ* term of Eq. (5). The *VD* term consists in the product of two terms tending to zero quadratically as y and z increase. This integrand thus quickly converges to zero when evaluated out of the vortical structures of the wake but can not be considered as compact.

In the case of periodic wing kinematics (and thus forces), averaging Eq. (3) or (4) over a flapping period cancels the time-derivatives terms. Therefore, the average of the momentum flux results in the average force if the effect of the pressure on the boundaries of the control volume is neglected, which is an acceptable assumption for a large control volume.

The expression given in Eq. (5) can be evaluated using measurements of the velocity in a single, sufficiently large outflow plane behind the bird. From the developments of this section, we see that it enables the estimation of both the average and the unsteady circulatory lift of a bird. Next section describes two numerical experiments designed to verify the validity of the estimation provided by Eq. (5).

2.5 Experiments to apply the lift estimation models

Two numerical experiments are reported in this paper, namely experiment 1 and experiment 2. In both experiments, the ground-truth velocity and vorticity fields are available over the whole simulation domain.

In experiment 1, the bird displays perfectly periodic wing kinematics guaranteeing stationary flight. That is, the total forces and moments acting on the bird average to zero over the flapping period. This experiment is used to evaluate how accurately the expression of the lift of Eq. (5) approaches the actual lift of the bird. Equation (5) is evaluated for outflow planes at various distances downstream from the bird.

We use the data collected in experiment 1 to evaluate the average of the lift estimation as a function of the position of the outflow plane. It is computed through a time integration with the trapeze method of the signal obtained with Eq. (5). This allows us to evaluate the contribution of each term of Eq. (5) to the average vertical force~~,~~ and their relevance when one is interested in the average weigh support.

As stated in section 2.4, the estimation provided by Eq. (5) is delayed with respect to the instantaneous lift. The ~~time-delay~~ between the output of Eq. (5) is evaluated for this periodic flight. At each downstream position, it is computed by shifting the estimation provided by Eq. (5) through time with a delay Δt comprised between 0 and the flapping period. For each value of Δt, the shifted result is correlated to the actual instantaneous lift ~~function~~ and the value of Δt maximizing this correlation is selected. To the ensemble of the best values of Δt at each downstream distance, we finally apply a linear regression in order to get a coefficient a capturing this relationship as

$$\Delta t = a x_{out} \ . \tag{6}$$

In experiment 2, the bird uses kinematics that ~~lead~~ to non-periodic lift. We use the data collected in this experiment to test the validity of the lift estimation when the period-averaged lift varies.

To obtain the kinematics, we use a closed-loop controller stabilizing the bird. Details about this controller are reported in [12]. After 5 seconds of level flight, the bird is tasked to change its lift so that it follows the following equation

$$L_{\text{targ}} = L_0 \sin\left(2\pi\left(t-5\right)/10\right) \ , \tag{7}$$

where $L_0 = 5$ N is an arbitrary force. The lift thus varies for 10 seconds. After those 10 seconds, the controller determines the target lift in order to stabilize the bird at a constant altitude. During the manoeuver, the controller also ensures that the bird remains at a constant streamwise velocity.

This results in variations of the lift, which is no longer periodic. The estimations are obtained in this case with the KJ term only, and shifted through time using $\Delta t = (x_{out} - x_{bird})/U_\infty$, where x_{out} and x_{bird} are the respective positions of the outflow plane and the bird. While the outflow plane is fixed, the bird can move through the domain. Its position should be evaluated at a time $t - \Delta t$ to ensure that the delay is correctly evaluated. ~~It however~~ constitutes an implicit equation. In this experiment, we compute Δt with a position evaluated at a time $t - \Delta t_0$, where Δt_0 is obtained using the initial position of the bird.

3 Results

The results from the simulations and the application of the lift estimation techniques presented in section 2.4 are reported in this section.

Figure 3 shows slices of streamwise vorticity ω_x with vectors representing the velocity in the plane. The velocity and vorticity fields are measured one wavelength ($\lambda = U_\infty/f$) of the wake behind the bird during experiment 1 (periodic and steady flight). We notice that the streamwise vorticity is lower at the beginning of the downstroke

than in the other phases. This implies that the circulation of the wing reaches a minimum around that time. During the downstroke, strong tip and root vortices are easily identified. The root vortex can be split into two cores of the same sign, one due to the drop of circulation at the wing root and the other related to the gradient of circulation along the wing due to the flapping motion. In the upstroke, the tip vortex changes sign, and a third vortex appears in the center of the wing due to the gradient of circulation.

Figure 3: Experiment 1: Four snapshots of the wake behind the bird. The snapshots are taken when the wing is mid-downstroke (A), at its downmost position (B), mid-upstroke (C), and in the topmost position (D). At each instant, the magnitude of the vorticity field is rendered in 3D, and the streamwise component of the vorticity is shown in a cross-flow slice situated one wavelength ($\lambda = U_\infty / f$) behind the bird. The arrows represent the velocity in the plane.

The total vertical aerodynamic force acting on the bird is showed in Figure 4a. The start of the represented flapping period corresponds to the middle of the downstroke. The exact lift based on the aerodynamic performances of the ILDL is compared with the evaluation of Eq. (4), showing good agreement. Although the added-mass effect is not accounted for in the simulation, an *a posteriori* estimation of the related contribution, using Eq. (1), is also represented in Fig. 4a.

(a) Total lift and added mass

(b) Decomposition of the terms

Figure 4: Experiment 1: Subfigure (a) shows the total vortex lift obtained through the ILDL (dashed black) and using Eq. (4). The red curve corresponds to an estimation of the added mass force. Subfigure (b) shows a decomposition of the terms of Eq. (4) into the corresponding terms of Eq. (3). Blue curve corresponds to the total, red to the momentum flux part, yellow to the external forces and purple to the time-derivative.

We observe from Fig. 4b that the simple evaluation of the momentum flux on a closed surface around the bird fails to recover the correct lift force – even with a delay. The term corresponding to the variations of momentum in the volume averages to zero in the case of periodic flight, but the one corresponding to the external forces (e.g. the variations of pressure on the boundaries) does not and contributes to the average lift.

Figure 5 shows the time evolution of the terms in Eq. (5) over a time corresponding to one flapping period and in four different outflow planes situated at increasing downstream distances.

(a) $x_{out}/\lambda = 0.25$

(b) $x_{out}/\lambda = 1$

(c) $x_{out}/\lambda = 1.75$

(d) $x_{out}/\lambda = 2.5$

Figure 5: Experiment 1: Evaluation of both terms of the lift estimation over time, for several downstream positions x_{out} of the outflow plane.

Although the terms of Eq. (5), which are the ones used in Fig. 5, derive from the momentum flux on the outflow plane, we observe in Fig. 5 that the estimation provided by Eq. (5) has similar values for the extrema and globally the same shape as the actual lift, which was not the case for the results pictured in Fig. 4. The quality of the estimation also degrades when the outflow plane moves further downstream from the bird. The phase information is not recovered when using Eq. (5) and the phase difference between the estimation and the actual lift varies with the distance.

We compute the delay as a function of the distance between the bird and the outflow plane, as explained in 2.5. Performing a linear regression on the values of the delay for several distances, we obtain a slope that is almost exactly equal to the free stream velocity, with a relative difference of $8.57\,10^{-5}$. Figure 6 shows the result of the time-shifting of the KJ estimations of Fig. 5.

Figure 6: Experiment 1: KJ estimations of the instantaneous lift for several positions of the outflow plane x_{out}, shifted in time with $\Delta t = x_{out}/U_\infty$.

The evolution of the average lift with the distance is also evaluated. As discussed in section 2.4, the time derivative term then drops. Figure 7 thus shows how the KJ estimation of the period-averaged lift varies with the position of the outflow plane and how the VD term affects it.

Figure 7: Experiment 1: Average the lift estimation (adimensionalized with the weight W of the bird) as a function of the downstream distance. Blue curve corresponds to the KJ term alone, and red curve to the sum of KJ and VD terms.

From the results shown in Fig. 7, we see that estimating the average lift of a flapping flier using only the KJ term provides a good approximation, with an error of less than 3% up to ten spans downstream. The VD term does not contribute much in the estimation and slightly increases the error of the estimation. This is however performed in a simulation without noise so we expect the error to be increased in actual experimental setups.

All the previous results are obtained in the setup provided by experiment 1, that is for a periodic steady flight. Figure 8 shows an estimation of the lift during experiment 2 (see section 2.5) with non periodic flapping cycles. The estimation obtained with the KJ term only is shifted through time with a delay $\Delta t = x_{out}/U_\infty$. Both the unsteady and period-averaged lift are estimated in three different outflow planes situated at $x_{out}/b = 1$, 4 and 7, and compared with the lift obtained from the ILDL.

Figure 8 shows that the error on the estimation of the lift increases with the distance between the bird and the outflow plane. The estimation of the unsteady lift reaches higher maximum values for increasing x_{out} and the average of the estimation is also higher than the reference provided by the ILDL-based aerodynamic performances by approximately 5%. However, despite the fact that the lift is not periodic, the phase information is correctly recovered with a time shift of $\Delta t = x_{out}/U_\infty$.

Figure 8: Experiment 2: Estimation of the lift for non-periodic flapping cycles. The upper figure shows the estimations of the instantaneous lift and the lower one of the lift averaged over the previous flapping period ($\bar{F}_z = \frac{1}{T}\int_{t-T}^{t} F_z \, \mathrm{d}t'$), for several distances x_{out} of the outflow plane. The force is expressed in Newtons in both figures.

4 Discussion

4.1 Simulated bird wake

The simulation framework allows reproducing the wake of a flying and flapping bird~~,~~ as if it was observed in a wind tunnel. The simulated wake shows similar structures to that of an actual bird. The most notable features are strong tip vortices, an expected feature in flapping or finite wing flight wakes [17, 18, 6]. The wake also presents root vortices that have been observed in [18, 6, 7] and spanwise vortices due to ~~time-variations~~ of the wing circulation, as in [4, 9].

During the upstroke, the tip vortex changes sign ~~and~~ a third vortex appears at a mid-span location along the wing. While these phenomena are not usually observed in bird flight, they have been ~~for~~ bat flight ~~in~~ [7].

4.2 Lift estimation

In Fig. 4a, we see that the evaluation of the full equation (4) agrees very well with the force from the lifting line. The ~~added-mass~~ ~~also~~ represented in Fig. 4a ~~is~~ of much lower amplitude than the total lift but is not necessarily negligible. When averaged, though, this contribution becomes negligible compared to the average lift.

As stated before, the momentum flux alone does not provide a good estimation for the total lift. Even when computed on a closed surface around the bird, it results in a force profile that is delayed and lower on average than the actual lift. In the results shown in Fig. 4, the control volume has a width equal to 6 times the span of the bird ~~and~~ the term related to the influence of the pressure is still significant.

Figure 5 shows that the KJ term gives ~~a~~ acceptable approximation of the lift − with a delay, as discussed in section 2.4. Although this term is derived from the flux of momentum through the outflow plane, the good agreement between the total force and this estimation implies that it is more accurate to use it than to directly integrate the momentum flux. This is due to the use of vorticity in Eq. (5) − which is a compact field − instead of the velocity (like in Eq. (4)) in the integrals giving an estimation of the lift. Thus, a finite outflow plane can provide an exact evaluation of the integrals~~,~~ as long as it encompasses the region of non-zero vorticity.

The contribution of the *velocity departure* term to the total lift estimation appears to be negligible. This means that access to the local streamwise velocity in the outflow plane is not necessary to obtain a good estimation of the lift~~,~~ as long as the free stream velocity is known. The fact that the plane of integration does not extend to

infinity implies that the evaluation of the term is incomplete (because the velocity does not form a compact field). However, since the contribution of the VD term is negligible, this is not an issue.

Wang and his co-authors used the KJ term to evaluate the lift of a flapping plate in [11]. As in the present work, they obtained good results when computing the average lift, in which case the ~~added-mass~~ contribution is deemed ~~as~~ negligible. This is backed by the similarity of the results for the average lift in this work and in [11]. The use of the vorticity in the outflow plane to estimate the instantaneous lift gave poor results in [11], even when correcting for the delay. These deviations with respect to the actual lift are possibly due to the contribution of adde[illegible]ass or viscosity effects~~,~~ since the Reynolds number they used was relatively low ($Re = 300$). Concerning the former, it can be seen in figure 12 of [11] that the amplitude of the ~~added-mass~~ lift is about twice that of the ~~vortex related~~ force. The magnitude of the added mass effect relative to the vortical lift is directly related to the reduced frequency k (see appendix B). In their case, this parameter was equal to 1.89, while in our simulations, the reduced frequency based on the mean aerodynamic chord of the wing is only 0.15. Therefore, it is expected that the added mass effects would be ~~a~~ tenfold more influential in their setting than in ours. Concerning the effect of viscosity, its importance is inversely proportional to the Reynolds number ~~and~~ it increases the streamwise diffusion of vertical momentum. The time-averaged momentum flux through an outflow plane is conserved, but its diffusion in the streamwise direction dampens the oscillations in the estimations of the lift, as clearly revealed by Figures 2, 9 ~~and~~ 16 of [11]. Since our simulations are performed at a higher Reynolds number ($Re \simeq 10^5$), this streamwise diffusion occurs more slowly and is actually overwhelmed by the self-induced distortions of the wake. Therefore, the unsteady lift can be recovered for further positions of the outflow plane before the distortions affect the instantaneous estimation. The framework of the present work is thus more adapted to the study of flight of large birds, ~~that~~ is characterized by lower values of the reduced frequency and higher Reynolds numbers.

In our simulations, we compute the delay between the instantaneous lift and the estimation obtained with the KJ term. This delay is related to the time necessary for the vorticity shed by the bird to reach the outflow plane. While this delay is likely directly related to the forward flight velocity of the bird V and evolving as $\Delta t = x_{out}/V$, it may be affected by the streamwise velocities induced by the bird and its wake. The fact that this delay evolves almost exactly as $\Delta t = x_{out}/U_\infty$ is linked to the horizontal equilibrium of the bird. Even though profile drag induces a velocity deficit in the wake, the wings produce an equal thrust force, accelerating the fluid. However, if the bird has a non-zero horizontal acceleration, its exchanges of momentum with the fluid may lead to the vorticity in the wake traveling faster or slower, and thus to an effect on the delay of the estimation.

In the second numerical experiment, the bird generates non periodic wing kinematics, leading successively to an increase and a decrease of the lift before settling to the initial value. We saw in this experiment that the variations of lift were smaller than expected. The flight dynamics indeed leads to vertical velocities of the bird that tend to reduce the variations of lift by affecting the angle of attack. In this case, Fig. 8 shows that the time shift corresponds to the time needed for the wake to travel to the measurement plane. Any changes in the average velocity at which the wake travels downstream are negligible here because the horizontal accelerations of the bird are very small.

Although the quality of the estimation remains acceptable at a distance of seven spans, we see that it degrades as the integration plane moves further downstream from the bird. This is due to growing distortion in the wake,

leading to different travel times for the vortical structures. The average of the estimation shows a larger departure from the ground truth in this experiment than in the stationary case. The error increases as the distance between the bird and the outflow plane increases. To avoid imprecisions in the estimation of both the instantaneous and averaged lift, as expected, the estimations are best performed at the closest possible position downstream from the bird.

5 Conclusion

In this paper, lift estimation techniques are applied to the simulation ~~of~~ bird flapping flight. The simulation framework mimics the conditions of a wind tunnel ~~and~~ the wake observed during the numerical experiments ~~present~~ the same structures as what has been observed in actual experiments [4, 6, 9].

The advantage of the numerical approach is that it provides access to the velocity and vorticity fields everywhere around the bird. It notably allows the evaluation of exact expressions of its lift based on velocity measurements, such as in Eq. (4). The result of the lift computed through this equation shows that we can indeed compute the instantaneous aerodynamic force provided that the velocity is accessible all around a bird. However, as discussed in the introduction, experimental works are more frequently based on measurements in a single plane in the wake of the bird. We have shown that it is possible to estimate the instantaneous lift of a flapping bird using ~~measurements of vorticity inside of~~ a cross-flow plane behind it.

We also identify the underlying assumptions ~~to~~ the estimation and quantify them. ~~Added-mass~~ is ignored in the estimation. The only way to properly evaluate its effect is to ~~access~~ the velocity all around the bird, but we find that its contribution is small in comparison to the circulatory lift. The ratio between the added mass force and the circulatory lift is approximately a quarter of the reduced frequency k, which is small for the studied case of large birds. In order to accurately recover the variations of lift, the viscous effects and the ensuing diffusion of momentum need to be negligible. We find it is the case for a Reynolds number of 10^5 corresponding to the flight of an ibis. Finally, for the estimation to be accurate, the bird needs to fly at a constant velocity without accelerating. When these conditions are met, we find that the average of the estimation exhibits remarkable consistency with increasing downstream distances ~~and~~ the proposed formula can also be used to recover instantaneous lift values, provided that a ~~time-delay~~ is accounted for.

Acknowledgments

Victor Colognesi is supported by a FRIA grant from the Fonds de la Recherche Scientifique de Belgique (F.R.S.-FNRS).

This work is part of a project supported by the ARC program of the Federation Wallonie-Bruxelles (grant number 17/22-080, RevealFlight).

Computational resources have been provided by the Consortium des Equipements de Calcul Intensif (CECI), funded by the Fonds de la Recherche Scientifique de Belgique (F.R.S.-FNRS) under Grant No. 2.5020.11 and by the Walloon Region.

The present research also benefited from computational resources made available on the Tier-1 supercomputer of the Federation Wallonie-Bruxelles, infrastructure funded by the Walloon Region under the grant agreement 1117545.

References

[1] Anders Hedenström. Extreme endurance migration: what is the limit to non-stop flight? *PLoS biology*, 8(5):e1000362, 2010.

[2] Robert E Gill Jr, T Lee Tibbitts, David C Douglas, Colleen M Handel, Daniel M Mulcahy, Jon C Gottschalck, Nils Warnock, Brian J McCaffery, Philip F Battley, and Theunis Piersma. Extreme endurance flights by landbirds crossing the pacific ocean: ecological corridor rather than barrier? *Proceedings of the Royal Society of London. Series B: Biological Sciences*, 276(1656):447–457, 2008.

[3] GR Spedding, Anders Hedenström, and Mikael Rosén. Quantitative studies of the wakes of freely flying birds in a low-turbulence wind tunnel. *Experiments in Fluids*, 34(2):291, 2003.

[4] GR Spedding, Mikael Rosén, and Anders Hedenström. A family of vortex wakes generated by a thrush nightingale in free flight in a wind tunnel over its entire natural range of flight speeds. *Journal of experimental biology*, 206(14):2313–2344, 2003.

[5] P. Henningsson, G. R. Spedding, and A. Hedenström. Vortex wake and flight kinematics of a swift in cruising flight in a wind tunnel. *Journal of Experimental Biology*, 211(5):717–730, 2008.

[6] P. Henningsson, F. T. Mujires, and A. Hedenström. Time-resolved vortex wake of a common swift flying over a range of flight speeds. *Journal of The Royal Society Interface*, 8(59):807–816, 2010.

[7] Florian T. Muijres, L. Christoffer Johansson, Melissa S. Bowlin, York Winter, and Anders Hedenström. Comparing aerodynamic efficiency in birds and bats suggests better flight performance in birds. *PloS one*, 7(5):e37335, 2012.

[8] Marco KleinHeerenbrink, Kajsa Warfvinge, and Anders Hedenström. Wake analysis of aerodynamic components for the glide envelope of a jackdaw (corvus monedula). *Journal of Experimental Biology*, 219(10):1572–1581, 2016.

[9] L Christoffer Johansson, Masateru Maeda, Per Henningsson, and Anders Hedenström. Mechanical power curve measured in the wake of pied flycatchers indicates modulation of parasite power across flight speeds. *Journal of The Royal Society Interface*, 15(138):20170814, 2018.

[10] E. Gutierrez, D. B. Quinn, D. D. Chin, and D Lentink. Lift calculations based on accepted wake models for anumal flight are inconsistent and sensitive to vortex dynamics. *Bioinspiration & Biomimetics*, 12(1):016004, 2016.

[11] Shizhao Wang, Guowei He, and Tianshu Liu. Estimating lift from wake velocity data in flapping flight. *Journal of Fluid Mechanics*, 868:501–537, Feb. 2019.

[12] Victor Colognesi, Renaud Ronsse, and Philippe Chatelain. Model coupling biomechanics and fluid dynamics for the simulation of controlled flapping flight. *Bioinspiration & Biomimetics*, 16(2), jan 2021.

[13] Denis-Gabriel Caprace, Grégoire Winckelmans, and Philippe Chatelain. An immersed lifting and dragging line model for the vortex particle-mesh method. *Theoretical and Computational Fluid Dynamics*, 34:21–48, 2020.

[14] Gavin K. Ananda and Michael S. Selig. Design of bird-like airfoils. In *2018 AIAA Aerospace Sciences Meeting*, page 0310, 2018.

[15] Mark Drela. Xfoil: An analysis and design system for low reynolds number airfoils. In *Low Reynolds number aerodynamics*, pages 1–12. Springer, 1989.

[16] F. Noca, D. Shiels, and D. Jeon. A comparison of methods for evaluating time-dependent fluid dynamic forces on bodies, using only velocity fields and their derivatives. *Journal of Fluids and Structures*, 13(5):551–578, Jul 1999.

[17] G. R. Spedding. The wake of a kestrel (falco tinnunculus) in flapping flight. *Journal of Experimental Biology*, 127(1):59, 01 1987.

[18] L. C. Johansson and A. Hedenström. The vortex wake of blackcaps (*Sylvia atricapilla* l.) measured using high-speed digital particle image velocimetry (dpiv). *Journal of Experimental Biology*, 212(20):3365–3376, 2009.

A Derivation of the lift estimation formula from the momentum flux

In section 2.4, we transform the momentum flux term of Eqs (3) and (4) into a more compact form. We thus start from

$$\frac{\mathbf{F}_{momflux}}{\rho} = -\oint \hat{\mathbf{n}} \cdot \mathbf{u}\mathbf{u} \, \mathrm{d}S \; . \tag{8}$$

When considering only the lift component of Eq. (8), we get

$$-(-\hat{\mathbf{e}}z) \cdot \oint \hat{\mathbf{n}} \cdot \mathbf{u}\mathbf{u} \, \mathrm{d}S = \oint \hat{\mathbf{n}} \cdot \mathbf{u} \, u_z \, \mathrm{d}S \; . \tag{9}$$

If the closed surface used for the integration of Eq. (9) extends to infinity in the y and z directions and towards the inflow, the contributions of these surface will drop to zero as we discussed in section 2.3. The only remaining integral is thus the one on the plane normal to x in the outflow, where the normal is $\hat{\mathbf{n}} = -\hat{\mathbf{e}}_x$. We thus have

$$\oint \hat{\mathbf{n}} \cdot \mathbf{u} \, u_z \, \mathrm{d}S = -\int_{S_{out}} u_x u_z \, \mathrm{d}S \; . \tag{10}$$

Equation (10) can be separated into two components:

$$-\int_{S_{out}} u_x u_z \,\mathrm{d}S = U_\infty \int_{S_{out}} u_z \,\mathrm{d}S - \int_{S_{out}} (U_\infty + u_x)\, u_z \,\mathrm{d}S \ . \tag{11}$$

The first integral of Eq. (11) can be integrated by parts, giving finally

$$\int_{S_{out}} u_z \,\mathrm{d}S = \int_{S_{out}} \left(\frac{\partial}{\partial y} (y\, u_z) - y \frac{\partial u_z}{\partial y} \right) \mathrm{d}S = -\int_{S_{out}} y \frac{\partial u_z}{\partial y} = -\int_{S_{out}} \left(y\, \omega_x + y \frac{\partial u_y}{\partial z} \right) \mathrm{d}S = -\int_{S_{out}} y \omega_x \,\mathrm{d}S \ . \tag{12}$$

B Ratio of the circulatory to added mass lift forces for a 2D wing in plunging motion

Consider a plunging wing in 2D with a lift coefficient following

$$C_l = 2\pi\alpha \ ,$$

with α being the instantaneous angle of attack, and an added mass of

$$m_a = \rho\, \pi\, c^2/4 \ ,$$

with c being the wing chord. The vertical position of the wing results from a harmonic motion

$$z(t) = A \sin(\omega\, t) \ ,$$

with A being an amplitude and $\omega = 2\pi\, f$ the angular frequency of the plunging motion.

The instantaneous angle of attack and acceleration of the wing are respectively given by

$$\alpha = \mathrm{atan}\left(-\frac{\dot{z}}{U} \right) = \mathrm{atan}\left(-\frac{\omega\, A \cos(\omega\, t)}{U} \right) \ ,$$

and

$$\ddot{z} = -\omega^2 A \sin(\omega\, t) \ .$$

The vertical forces due to the effect of the angle of attack and to the added mass are thus expressed as

$$C_{l,\alpha} = 2\pi\, \mathrm{atan}\left(-\frac{\omega\, A \cos(\omega\, t)}{U} \right) \ ,$$

and

$$C_{l,am} = \frac{2 m_a\, \ddot{z}}{\rho\, U^2\, c} = \frac{\pi\, c}{2U^2} (\omega)^2 A \sin(\omega\, t) \ .$$

If the vertical velocity of the wing is small compared to the horizontal velocity U the atan can be simplified to its argument. In this case, the ratio of the amplitudes of both forces contributions is expressed as

$$\frac{C_{l,\alpha}}{C_{l,am}} = \frac{4\pi\, U\, \omega\, A}{\pi\, c\, \omega^2\, A} = \frac{4U}{\omega\, c} = \frac{2}{k} \ .$$

where $k = \frac{\omega\, c}{2U}$ is called the reduced frequency of the motion.

We can thus see that the ratio between these two contributions to the vertical force of a purely plunging airfoil is inversely related to the reduced frequency, and that the larger its value, the more important the added mass effects.

Figure 1

Click here to access/download;Figure;figure1.png

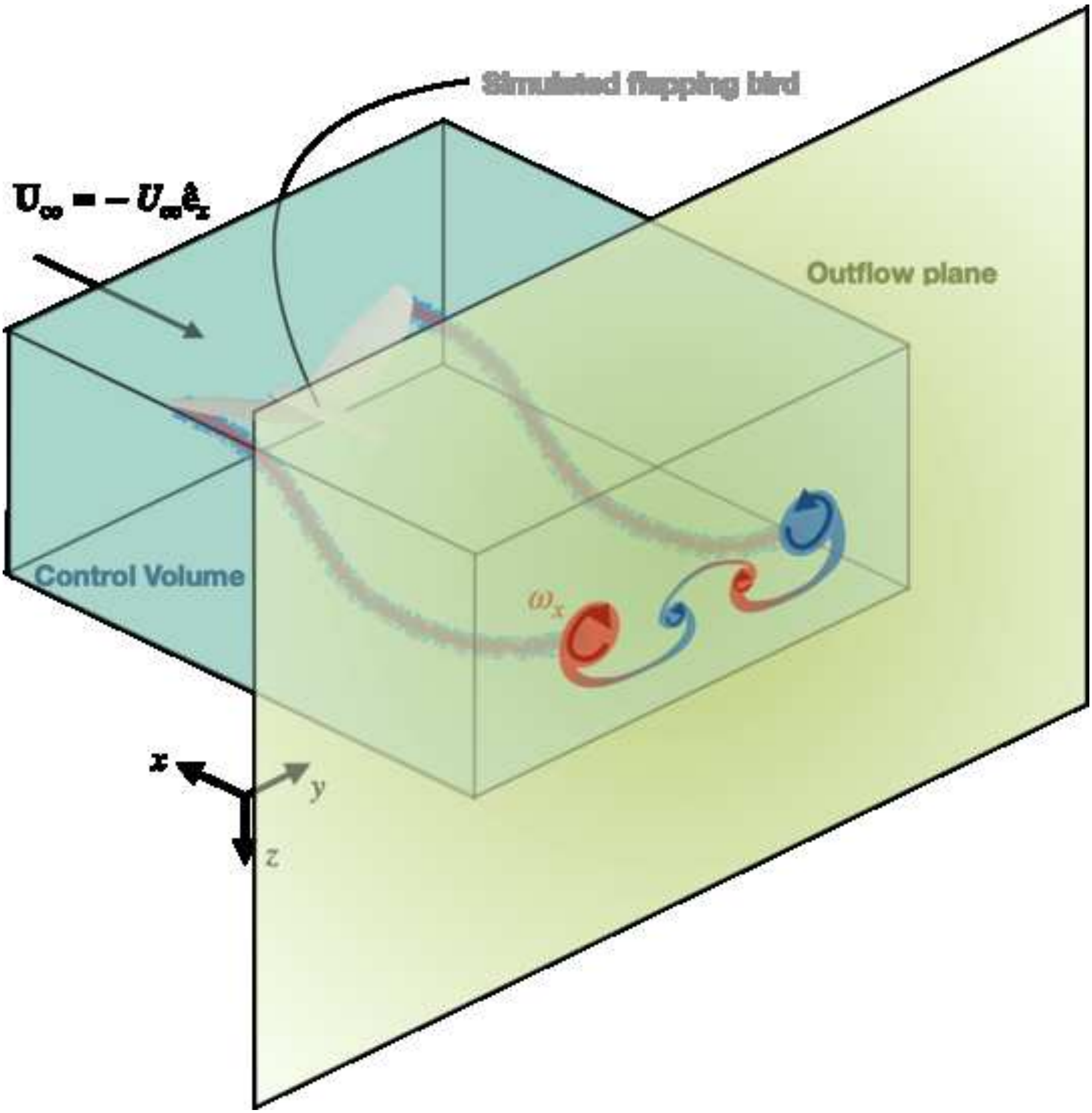

Figure 2

Click here to access/download;Figure;figure2.pdf

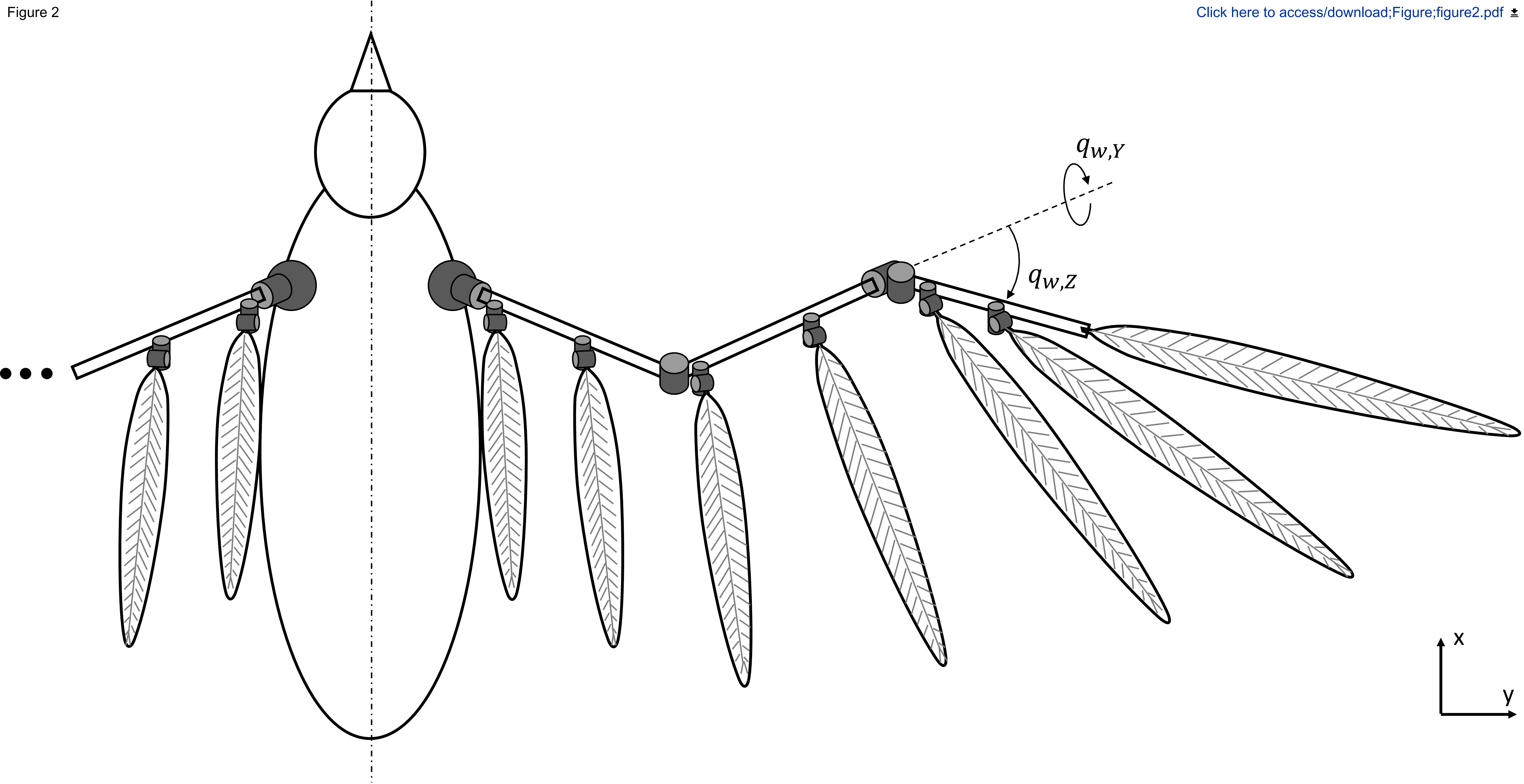

Figure 3

Click here to access/download;Figure;figure3.png

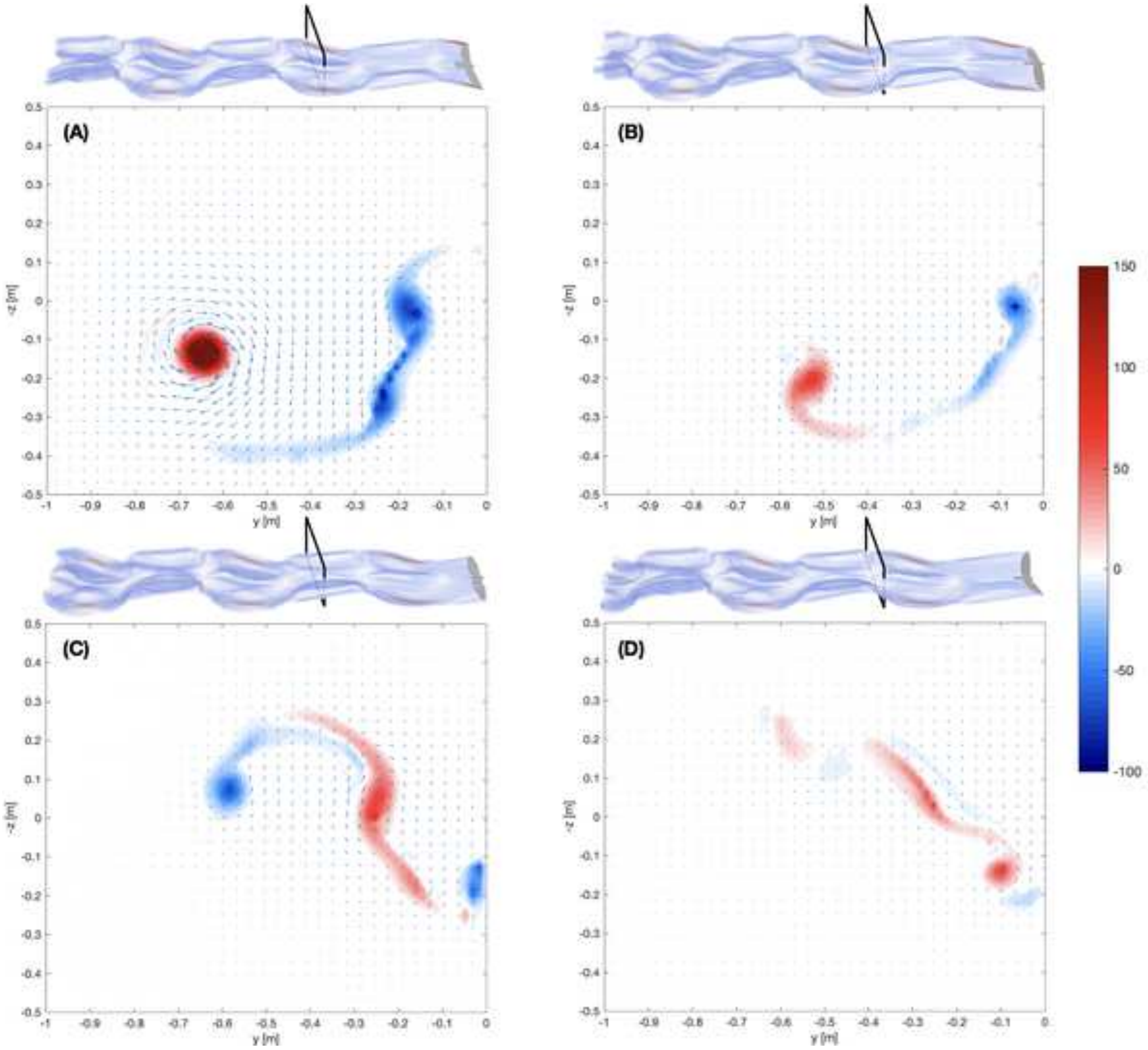

Figure 4a

Click here to access/download;

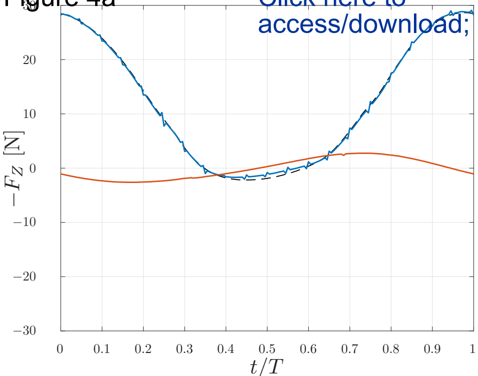

Figure 4b

Click here to access/download;

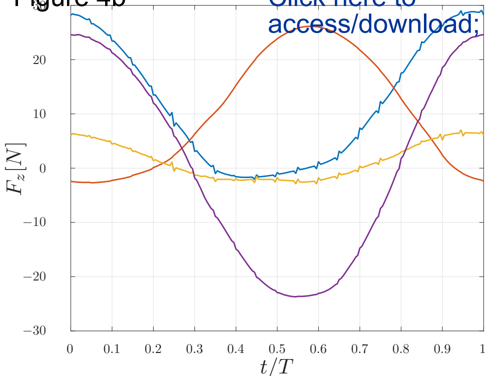

Figure 5a

Click here to access/download;

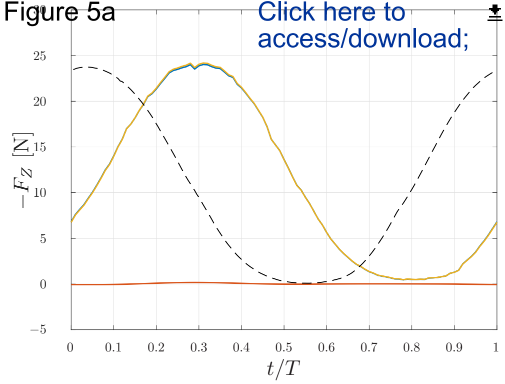

Figure 5b

Click here to access/download;

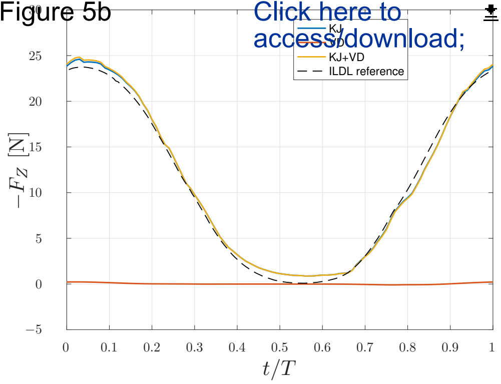

Figure 5c

Click here to access/download;

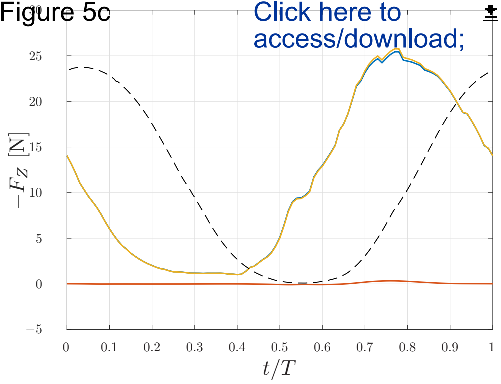

Figure 5d

Click here to access/download;

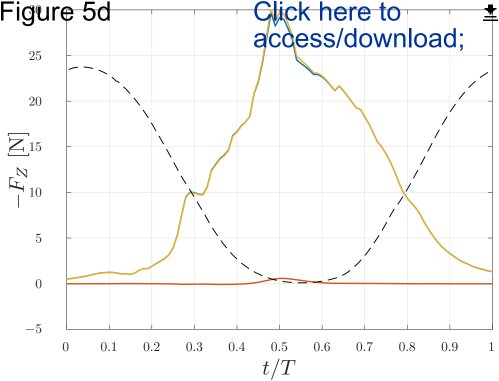

Figure 6

Click here to access/download;Figur

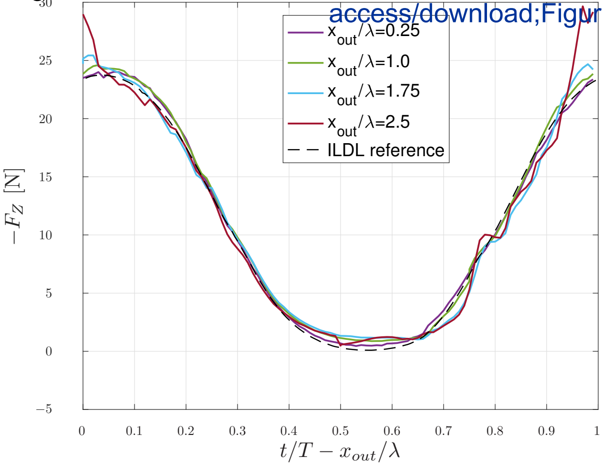

Figure 7

Click here to access/download;Figur

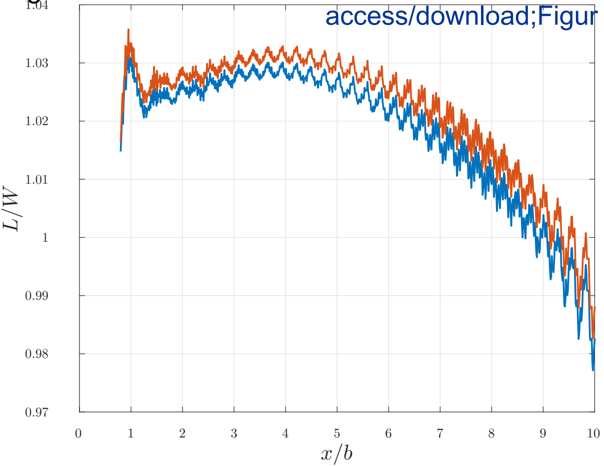

Figure 8

Click here to access/download;Figure;figure8.pdf

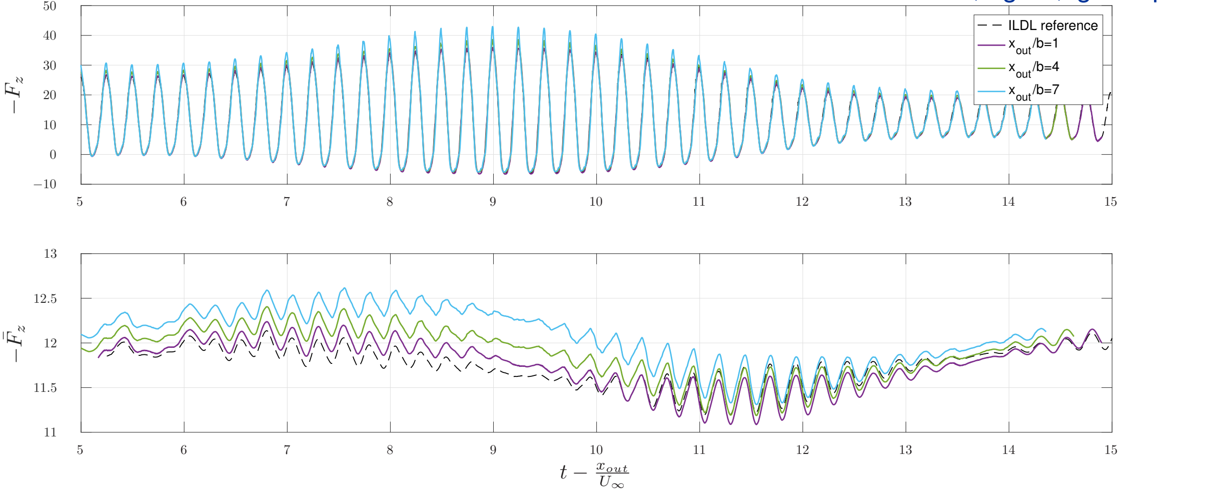

Data Review URL

Click here to download Data Review URL

http://dataverse.uclouvain.be/dataset.xhtml?persistentId=doi:10.14428/D

Data Review URL

Click here to download Data Review URL

http://dataverse.uclouvain.be/dataset.xhtml?persistentId=doi:10.14428/D
