# Supplementary material for: Numerical assessment of wake-based estimation of instantaneous lift in flapping flight of large birds
Source: PLoS One. 2023 May 4;18(5):e0284714. doi: 10.1371/journal.pone.0284714 (PMC10159204; doi:10.1371/journal.pone.0284714)
Supplement: Attachment — Submitted filename: response_to_reviewers.pdf [file pone.0284714.s007.pdf]

Numerical assessment of wake-based estimation of instantaneous lift in flapping flight of large birds – reply to reviewers

Victor Colognesi, Renaud Ronsse, Philippe Chatelain
UCLouvain; Institute of Mechanics, Materials and Civil Engineering
Louvain-la-Neuve, Belgium

January 27, 2023

Introduction

First and foremost, we wish to thank the reviewers for their time and careful review of the work. Their remarks greatly helped us improve the quality and increase the scope of the manuscript.

Throughout this document, all text taken from the manuscript will be written in *italic*, and references are numbered as in the manuscript. In the revised version of the manuscript, the changes related to remarks of Reviewer 1 are written in green, the ones related to remarks of Reviewer 2 in blue, the changes regarding both reviewers are marked in purple, and the changes in red correspond to corrections of typos and grammatical mistakes.

1 Remarks common to both reviewers

In this first section, we address the remarks and questions that were common to both reviewers.

1.1 Improved explanation and description of the numerical method and setup

The first major remark of Reviewer 2 pointed out that the numerical setup was not fully detailed, and that it was necessary to complete its description in the methods section. Other remarks from both reviewers pointed to missing information or required changes in the description of the methods.

We thus substantially reworked section 2.1 of the manuscript to include more detail regarding the numerical setup. The reworked section is itself divided in subsections describing respectively the bio-mechanical model, the aerodynamic model, the flow solver and the actuation and control of the bird model.

We notably replaced figure 2 of the initial manuscript with a more detailed figure, including a representation of the actual bird wing (see Remark 3 of Reviewer 1), a 3D representation of the simulated bird (see minor remark 7 of Reviewer 2), and highlighting the positions of the joints of the skeletal model (see minor remark 13 of Reviewer 2).

Aside from the restructuring of the text and the addition of a figure, a lot of paragraphs have been added to this section. These will be discussed in the following sections to answer the relevant comments.

1.2 Justification of the choice of airfoil

Both reviewers shared concerns with the airfoil used in the simulations (see major remark 2 of Reviewer 1 and minor comment 9 of Reviewer 2). We agree that this airfoil, though bio-inspired, does not necessarily represent the wing profile of the considered species (*Geronticus Eremita*) with great accuracy. However, it still produces aerodynamic forces that are similar to what this bird would produce.

The main message of the manuscript regards the methods enabling an estimation of the aerodynamic forces produced by a bird (or rather a bird-like flier in our case) from wake measurements. We agree that the points raised by the reviewers lead to modeling errors at the level of the wing profile and would affect the aerodynamic forces. However, a starting assumption of our work is that the unsteady force distributions are given and biologically relevant (as opposed to a high-fidelity reproduction of a precise species and gait); the main goal of the paper is then to present methods to retrieve them. As the wake is produced in a manner which is consistent with the simulated aerodynamic forces, we believe that our computational framework enables the rigorous assessment of the validity of such techniques, even if the computed aerodynamic forces do not exactly correspond to those that an Ibis would produce.

This discussion has been summarized in the revised paper:

At each section of the wings, the lift and drag are computed based on the local instantaneous relative velocity, using polar data, i.e., the values of the lift and drag coefficients of a 2D profile as a function of both the local angle of attack and Reynolds number. The lift and drag coefficients at each point of the ILDL are those of an AS6092 airfoil [20]. It is a bird-like airfoil, and its polar is available in [20] and represented in appendix A. This particular airfoil has been chosen among the proposed airfoils of [20] for its limited camber and intermediate thickness. The polar of the airfoil is computed in [20] using XFOIL [21]. The aerodynamic forces computed along the ILDL take in consideration the effects of local chord, angle of attack, thickness and camber through the polar.

The use of a single airfoil for the whole bird wing is a major assumption, since camber and relative thickness realistically vary along the span of the bird. However, we believe that the aerodynamic forces computed with this assumption remain realistic – although they do not correspond exactly to the ones that the actual bird would produce. As our thesis rather lies in proving the recovery of the aerodynamic forces produced by the bird from wake measurements, a slight lack of fidelity in the generation of these forces does not undermine that core message, as long as they resemble the ones produced by an actual bird.

Moreover, since the bird is trimmed through the use of a controller, any changes in the polar would result in different kinematics, so that the bird is in equilibrium on average. This implies that departures from reality in the polar behavior (i.e., in the relationships between lift, drag and moment coefficient and the local angle of attack) will eventually lead to the same average forces, and only in different force distributions both through time and along the wingspan.

Experimental data on these aerodynamic force distributions is extremely difficult to find. At best, we can compare the wake resulting from them to bird wakes measured experimentally. Such comparison has been added in the results of the paper to attest to the biological relevance of our results.

1.3 Additional results and comparison with experimental data

Remarks from both reviewers pointed to a general lack of results about the bird aerodynamics and the resulting wake. The initial intent of the paper was to focus on the lift estimation techniques used to retrieve the aerodynamic forces from wake measurements, but we agree that improving the description and discussion of the wake results is important and improves the paper generally.

As a result, we added a new section in the results named *Aerodynamics and simulated wake* where we describe the aerodynamic forces and wake results obtained from our simulations. In these, we include a visualization of the distribution of the aerodynamic forces through time and along the wingspan, a 3D visualization of the wake under several angles (Fig. 4 of the revised paper), and 2D visualizations of the vorticity field in longitudinal (Fig. 5) and cross-flow (Fig. 6) planes.

This gave us the occasion of extensively describe the structure of the simulated wake, discuss it, and compare our results with experimental data, notably the measurements made in the wake of a swift by Henningsson *et al.* in [5] and [9]. In the discussion, we particularly compare our results to those showed in Fig. 4 of [5], which is represented in Fig. 1 of the present document.

This comparison shows that the obtained wake has a realistic structure for the high-aspect ratio bird of our simulations. We indeed find that the spanwise vorticity strongly resembles the one observed for the swift, and deforms in a similar way. The comparison of the vorticity at the level of the body is however different.

The presentation of these results and their comparison with the experimentally measured wake of another high-aspect ratio bird should further support the validity of our results. Indeed, even if the vorticity field used to assess

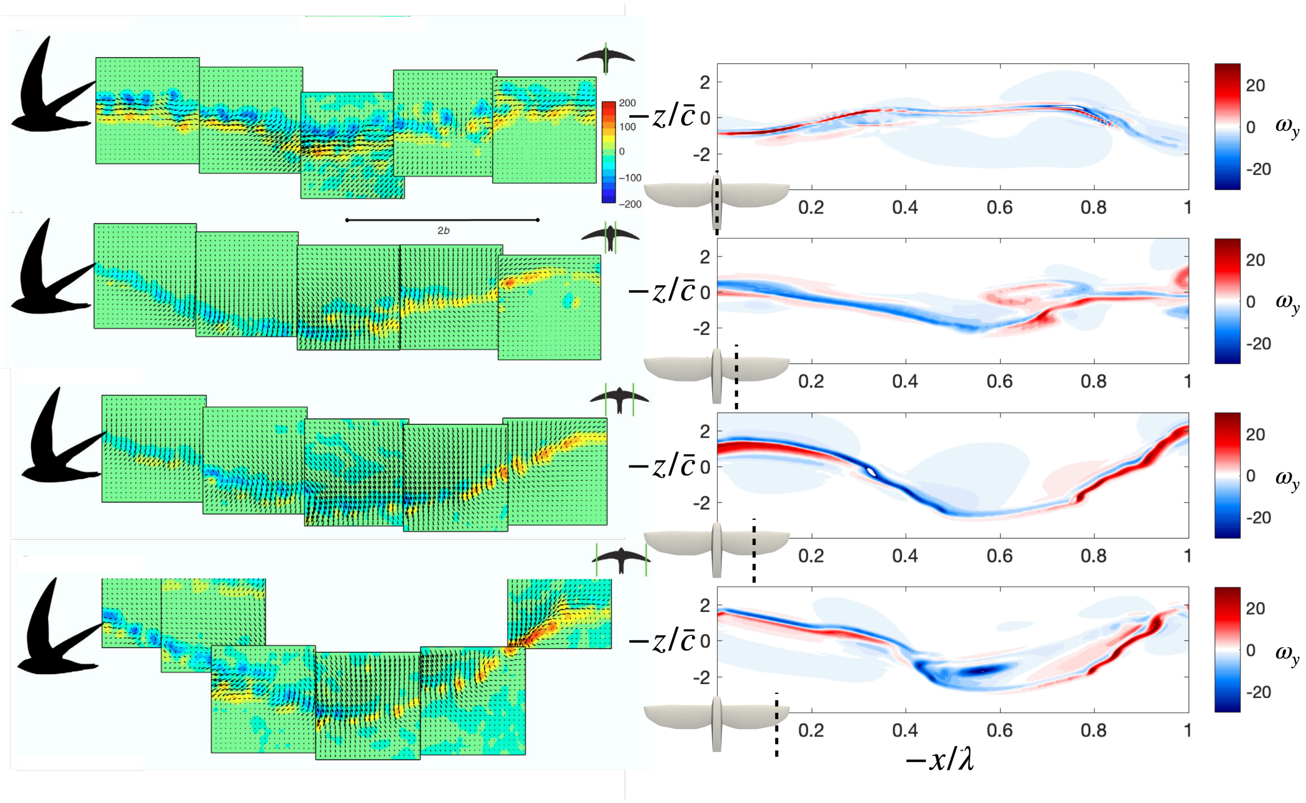

Figure 1: Comparison between our results and the ones of Henningsson *et al.* [5]

the lift estimation expressions is not exactly that of a precise bird species because of modeling errors, it is at least qualitatively accurate. Figure 1 further attests to the biological relevance of those vorticity fields; this relevance and our validated numerical framework are in the authors' opinion sufficient to validate the findings of our paper.

2 Reviewer 1

We thank Reviewer 1 for his interesting remarks. Modifications in the text that are related to his remarks are written in green. Modification regarding both reviewers or intended to clarify the message independently of remarks are written in purple. We took the liberty to separate the remarks of the reviewer that resulted in minor changes of the manuscript in a separate section. Thus, section 2.1 reports the most important comments, and their respective answers, and section 2.2 does the same for the lighter corrections.

2.1 Major remarks

Remark 1: This study derives and then validates an instantaneous lift equation using numerical simulation data of a flapping flight model. However, no validation data for the numerical solver had been provided. At the least, velocity profiles and vorticity profiles at the Trefftz planes (using the numerical solver) need to be compared with the experimental data. Although the authors provided a reference for the solver details, this comparison needs to be shown in the main manuscript. Without these comparisons with the experimental data, the validity of the solver remains questionable.

We understand the concerns of Reviewer 1 regarding the validity of our results. It is true that our model has never been properly validated with experimental data. However, in [16], we compare the power consumption of the bird estimated by our model to three other estimations (one experimentally measured, one extrapolated from experimental data of other birds, and one from another model), and our results are in the same order of magnitude

as the other references. The fluid solver used to model the wing aerodynamics, the shedding of vorticity and the evolution of the wake, has also been compared to experimental data for the flight dynamics of rotorcraft in [26], where it showed satisfactory agreement to the reality. Both of these comparisons are now mentioned in the methods section of the revised paper:

The momentum in the wake has been confirmed to correspond to the actual aerodynamic forces produced by a simulated bird in [16], and for fixed wings in [19]. This correspondence is key in our goal of deriving expressions for the estimation of lift from wake measurements. The model used to simulate the wing aerodynamics, the shedding of vorticity and the wake evolution has been compared to experimental data in the challenging case of rotorcraft in [26], where the aerodynamic forces and their variations with the control parameters of the rotor were accurately modeled. The coupled bio-mechanical and aerodynamic model used to simulate bird flight have been compared to reference results regarding flight power in [16].

Additionally, as mentioned in section 1.3, we complemented the paper with a description of the obtained wake, and a comparison with experimental data for another high-aspect ratio bird (from [5] and [9]). As the comparison shows that our wake model predictions are in a very close agreement with the experiments, we consider that it is relevant to apply the lift estimation techniques to our simulated wake.

Remark 2: The authors mentioned that AS6092 (bird-like) airfoil was used for the simulations. However, the simulated bird was Geronticus Eremita. This fact largely undermines the validity of this study as a slight difference in camber or curvature (with the actual bird airfoil) would produce significant difference in numerical simulation compared to the experimental data.

We agree that the airfoil used in our simulations does not necessarily represents the one of the simulated species with perfect accuracy. However, as discussed in section 1.2 of this document, we believe that our main message remains valid even for slightly different aerodynamic forces.

Remark 3: Again, birds' airfoil can vary significantly along the span in terms of camber and chord length. The authors didn't mention anything about the differences between the model and the actual bird (Geronticus Eremita). A figure showing the wing model as well as the actual bird wing is required to be added in the manuscript.

We thank the reviewer for pointing out that there was no illustration of the wings of the simulated bird. Figure 2 of the revised paper (reproduced in Fig. 2 of this document) now shows the 3D shape represented by the ILDL in the fluid solver. The wing represented in Fig. 2 and used in the simulations through the ILDL does have a varying chord. The simulation framework could also account for varying camber through a parameterized polar, albeit this is beyond the scope of this paper.

Remark 4: Also, this study lacks content. I would expect the authors to include some contents that show variations of lift as a function of the AOA. Since this is a numerical experiment, this can be done after validating the solver data with the level flight of the bird (experimental data).

We added an appendix to the revised paper describing the polar used in the simulations, and showing how its lift coefficient evolves with the instantaneous angle of attack. We also followed the suggestion of the reviewer and added a description of the aerodynamic forces produced by the bird. As discussed in section 1.3, Fig. 3 of the revised manuscript shows both the spanwise distribution and the evolution through time of the angle of attack, the lift coefficient, and the vertical and horizontal forces.

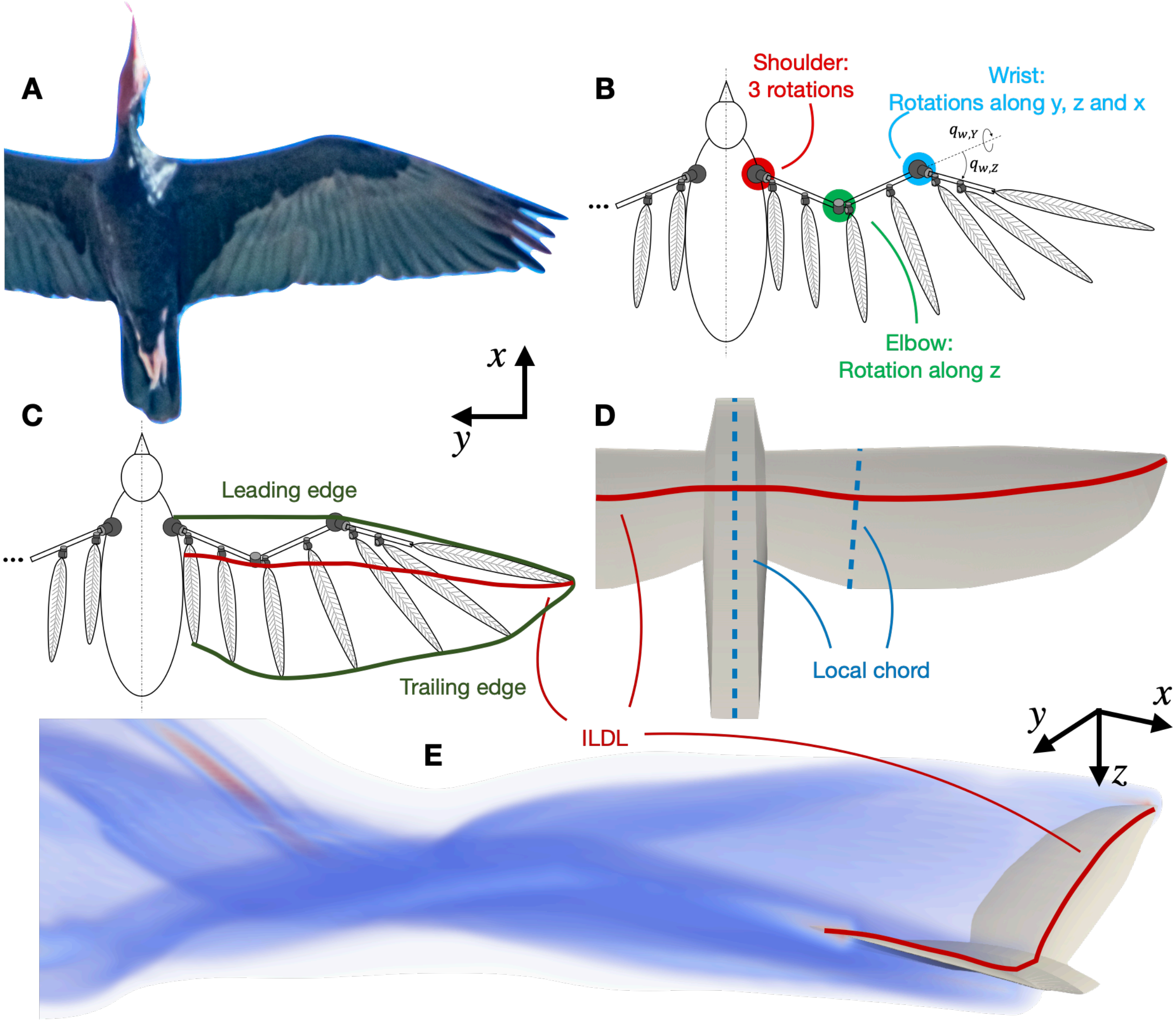

Figure 2: Various models used to represent the bird in the simulations. (A): Picture of the simulated bird gliding. (B) a representation of the bio-mechanical model of the skeleton and feathers, with the joints highlighted and two rotations of the wrist represented as examples. (C) a representation of the ILDL obtained in a particular configuration. (D) the final planform represented in the fluid solver and (E) the simulated bird and its near wake.

Remark 5: I would recommend authors to add more contents/pictures in Figure 3 (evolution of wake vortices). As this is a numerical simulation, the authors should be able to provide a much more detailed picture of evolution of vorticity at the wake.

We thank the reviewer for this suggestion. As discussed in section 1.3 of this document, we added more content in the results section.

2.2 Minor Comments

Comment 1: It is not clear to me how mathematical operations for the surface integral of the velocity were done to turn it into a surface integral of vorticity*distance (equation 12 in appendix). Can you please show the step by step processes?

The mathematical steps between the equalities of equation 12 (of the inital manuscript) have been explicitly showed in equations 12 to 15 of the revised manuscript.

Comment 2: The figures (4a-5d, 7) need line legend.

Line legends have been added to the figures.

Comment 3: To use the lift equation (equation 5), is there any requirement to use threshold on the vorticity maps? Did the authors use any threshold on Figure 3?

We evaluate the equations using the unfiltered velocity and vorticity fields, and, hence, we do not use any threshold. This has been precised in the results section:

Figure 8 shows the time evolution of the terms in Eq. (5) *over a time corresponding to one flapping period and in four different outflow planes situated at increasing downstream distances. The terms of the equation are evaluated with the unfiltered velocity and vorticity fields obtained in scenario 1.*

---

3 Reviewer 2

We thank Reviewer 2 for his meaningful comments and for the grammatical suggestions in the text. Modifications in the text that are related to his remarks are written in dark blue. Modification regarding both reviewers or intended to clarify the message independently of remarks are written in purple. Section 3.1 reports the major remarks of Reviewer 2 and their respective answers, and section 3.2 lists their minor comments and the corresponding changes to the manuscript.

3.1 Major remarks

Remark 1: The numerical setup utilized in the current paper is not detailed fully, omitting large portions that are relevant for the reader to follow. Here are a few parts that need to be adequately described in the paper: bird modeling, discretization, computational domain, numerical schemes, fluid/motion coupling, etc.

We agree that the methodological aspects were not properly described in the paper. The methods section has thus been significantly expanded, as discussed in section 1.1 of the present document.

Remark 2: The current version of the paper ignores a substantial portion of existing literature in the context of instantaneous lift estimation from the near-wake measurements behind flapping birds relevant to the present study. Here are a few examples that computed the unsteady drag and lift forces of freely flying birds, worth mentioning in the paper:

[1] Ben-Gida H., Kirchhefer A. J., Taylor Z. J., Bezner-Kerr W., Guglielmo C. G., Kopp G. A.,and Gurka R. (2013) “Estimation of unsteady aerodynamics in the wake of a freely flying European starling (Sturnus vulgaris)”, PLOS ONE 8(11), p. e80086. DOI: https://doi.org/10.1371/journal.pone.0080086

[2] Stalnov O., Ben-Gida H., Kirchhefer A. J., Guglielmo C. G., Kopp G. A., Liberzon A., andGurka R. (2015) "On the estimation of time dependent lift of a European starling (Sturnus vulgaris) during flapping flight", PLOS ONE 10(9), p. e0134582. DOI: https://doi.org/10.1371/journal.pone.0134582

[3] Nafi A., Ben-Gida H., Guglielmo C. G., and Gurka R. (2020) "Aerodynamic forces actingon birds during flight: A comparative study of a shorebird, songbird and a strigiform", Experimental Thermal and Fluid Science 113, p. 110018. DOI: https://doi.org/10.1016/j.expthermflusci.2019.110018

We apologize for missing these key contributions to the field and thank the reviewer for sharing these articles. As the first reference mainly focuses on estimations of the drag, we did not discuss it in the manuscript, but we properly included references to the latter two articles in the introduction:

Stalnov et al. *[6] and Nafi* et al. *[7] use a similar approach to estimate the lift of a bird, but limited to a single plane of measurement in its wake, which can not capture the spanwise variations of the wing circulation.*

Remark 3: Since the paper analyzes the case of a large flapping bird flying at a relatively high Reynolds number, I suggest the authors change the paper title to: "Numerical assessment of wake-based estimation of instantaneous lift in flapping flight of large birds".

We agree with the title proposed by the reviewer, as it describes more precisely the scope of validity of our study. We changed the title in the revised manuscript.

3.2 Minor Comments

Comment 1: In the Introduction, lines 25-26, pp. 1: "In order to experiment on a flying bird without hindering it with sensing equipment, one often relies on measurements in the wake of the animal." – Please re-write the sentence to clarify your meaning. What does it mean "to experiment on a flying bird"?

We apologize for the unclear wording. We modified the sentence referred by the reviewer to:

Collecting experimental data on the aerodynamic forces produced by a bird in flapping flight without hindering it with sensing instruments, often relies on measurements in the wake of the animal.

Comment 2: In the Introduction, lines 32-33, pp. 2: "Usually, circulation is considered to be zero at the start of the downstroke but this remains an estimation." – This statement is somewhat misleading. What circulation do you refer to? The total circulation generated by the bird? Circulation generated by the wings? Circulation generated by the tail? The circulation value could be different depending on the integration area of the vorticity field. Please revise the sentence to be more specific. Also, please present a reference for your statement.

We agree that this statement was misleading. We modified it to:

These models can be based on various assumptions. In the works of Spedding et al. [4] and Henningsson et al. [5], for example, the spanwise vorticity is evaluated (using PIV in a transversal plane). More precisely, [4] relies on simplified models to link the vorticity to lift values, where the wake is considered to be composed of distinct vortices forming either two deforming tip vortices of constant circulation, or vortex rings. In [5], only the variations of lift through a flapping cycle are considered, since the spanwise vorticity is linked to the temporal variations of the wing circulation.

Comment 3: In the Introduction, lines 49-51, pp. 2: "In [11], Wang et al. simulated the flow around and behind flapping wings to evaluate the validity of a lift estimation method and propose a correction of said method that enables an accurate estimation of the average lift." – Please indicate what lift estimation method was studied by Wang et al. (2019).

We precised briefly in the revised text how Wang *et al.* estimate the aerodynamic forces from wake measurements:

In [15], Wang et al. simulated the flow around and behind flapping wings to evaluate the validity of a lift estimation method based on the application of the Kutta-Joukowski theorem, using the streamwise component of the vorticity field in cross-flow planes. They propose a correction of the method that enables an accurate estimation of the average lift, but their proposed estimation fails to recover the unsteady lift of a flapping wing.

Comment 4: In the Introduction, lines 54-55, pp. 2: "In these conditions, the viscous and added mass effects have a lesser impact and we expect the unsteady lift to be recoverable further in the wake." – Please provide references to support your statement of neglecting the added mass for your case of study. Also, please elaborate on why do you expect that the unsteady lift can be recoverable further in the wake"?

What we meant by this statement is that, since the flight of the bird simulated in this study uses different values of the Reynolds number and reduced frequency – and that these are respectively linked to the viscous and added mass effects –, we expected different results. We agree however that, at this point, it is too soon to mention that added mass is negligible and that the time-dependent lift can be retrieved further in the wake. This is also discussed later in the manuscript, in light of our results. We modified the paragraph to the following:

We apply this framework to the flapping flight of a large migratory bird. This case is characterized by a higher Reynolds number and a lower reduced frequency than in [15]. As both of these parameters have a significant impact on the wake dynamics and the wing aerodynamics, we expect our results to differ from the ones of [15]. In particular, the Reynolds number is linked to the importance of viscous effects in the flow: at higher Reynolds number, viscous effects are less important than inertial ones, and thus, wake dissipation is slower. The reduced frequency is directly related to the relative importance of the added mass contribution to the lift compared to the one of the circulation (see appendix C). For lower values of this parameter, we expect the aerodynamic forces to be dominated by circulatory lift, which leaves a trace in the wake behind the bird [4, 10]. It is worth testing whether, under these conditions, the unsteady lift can be accurately estimated from wake measurements.

Comment 5: In the Introduction, lines 58-61, pp. 2: "We finally assess the validity of the lift estimation techniques in numerical experiments using an in-house model of a flapping bird [12], which is based on a vortex particle-mesh method and faithfully simulates the unsteady wake of the bird over large distances." – What are numerical experiments? Please use different wording. In addition, please add a reference to the vortex particle-mesh method.

We apologize for the poor choice of words. We changed the sentence pointed out by the reviewer:

We then assess the validity of the derived formulation in numerically produced wakes using our in-house flapping bird model.

We also added a reference to the fluid solver but the second half of the sentence has finally been mover earlier in the introduction.

In the present paper, we use an in-house model of a flapping bird [16] based on a vortex particle-mesh method [17], which can faithfully simulate the unsteady wake of a bird over large distances.

Comment 6: The quality of Figures 1 and 3 is very poor. Please improve the quality of these figures to better capture the flow details.

As mentioned in section 1.3 of this document, the results section has been significantly expanded, and now includes more descriptions of the wake, over various angles.

Comment 7: In Section 2.1, lines 72-76, pp. 3: "The whole bird is represented as a single line, which is obtained by extracting one line for each wing and merging them together to account for the influence of the body. At each time-step, the wing lines are computed from the position of the bones and feathers embodied into the bio-mechanical model. They are defined as a line passing through the quarter of the chord – i.e. the line linking the leading and trailing edges – normal to it at each point. In the center, the chord length is set to the length of the bird body." – How did you model and account for the bird's body in your computation? Was it treated as a bluff body? Something else? Moreover, did you model the bird's head or tail? The tail, in particular, can significantly affect the overall lift of the bird [1,2]. If not considered in the computations, the authors would need to present a solid argument as to why they think the tail interaction with the wake is negligible. Generally, I suggest the authors include a more representative three-dimensional image of the modeled bird (instead or in addition to Figure 2), so it would be easier for the reader to follow the actual bird geometry studied here.

The bird body is considered to be part of the ILDL representing the whole bird in our simulations. It is thus represented as a single spanwise line, where aerodynamic forces are computed, and that acts as a source of vorticity in the fluid solver. We completed the description of this methodology in an effort to make it clearer:

The body-tail ensemble is included in the ILDL. Between the wings, the chord length is set to the length of the bird body. In this region, the aerodynamic performance of the airfoil is degraded in regarding the lift-to-drag ratio. To account for the larger contribution of the body to the drag of the bird than to its total lift, we use a degraded lift coefficient compared to the rest of the ILDL. When computing the lift coefficient of the body, we divide the value obtained from the polar by 3 in the simulations, while keeping the same drag coefficient. This value roughly corresponds to the increase of chord length between the wing root and the body, and thus reduces the spanwise variation of the circulation at that location. This is an arbitrary value, but the main source of aerodynamic forces being the wings of the bird, we assume that this should have a minor effect on the results.

Regarding the role of the bird tail, literature does not agree on it. Depending on the species, the flight configuration and the studies, it can increase the total lift of the bird, decrease it, or have a negligible effect. We added a paragraph to discuss this and justify why we neglect its impact on the aerodynamic forces:

The lift contribution of the body-tail ensemble in real birds is still a matter of open discussion. In [22], it is shown that the body-tail of gliding raptors have a higher circulation than the wing at their root, and thus produces more lift. However, in other experimental results comparing the wakes of flapping birds with and without a tail [23], no evidence was found for an aerodynamic effect of the tail. Finally, in [9], the effect of the tail was found to significantly vary with the flight velocity, with higher (resp. lower) lift produced at lower (resp. higher) velocities. Since our scenario concerns steady flapping flight at a high forward velocity, we consider that the assumption of low tail lift is reasonable.

Finally, we added a 3D image of the modeled bird in figure 2 of the revised paper (see Fig. 2 of the current document).

Comment 8: In Section 2.1, lines 79-81, pp. 3: "In the body region, the aerodynamic performance of the profile are degraded in terms of lift-to-drag ratio. We thus divide the lift coefficient by 3 in the simulations. This captures the higher contribution of the body to the drag than to the lift of the whole bird." –What do you mean by "We thus divide the lift coefficient by 3 in the simulations"? Please elaborate in the text to better describe your modeling approach of dividing the lift coefficient by 3, as the reasoning is unclear.

The polar used for the computation of the lift coefficient of the body is modified with respect to the rest of the bird, so that for a given angle of attack, the lift coefficient would be three times smaller. The value 3 by which we divide the lift coefficient is arbitrary, but corresponds roughly to the increase of chord length from the wing root to the body. We added this discussion in the manuscript, to better explain our reasoning:

To account for the larger contribution of the body to the drag of the bird than to its total lift, we use a degraded lift coefficient compared to the rest of the ILDL. When computing the lift coefficient of the body, we divide the value obtained from the polar by 3 in the simulations, while keeping the same drag coefficient. This value roughly corresponds to the increase of chord length between the wing root and the body, and thus reduces the spanwise variation of the circulation at that location.

Comment 9: In Section 2.1, line 82, pp. 3: "The profile used in these simulations is an AS6092 airfoil [14]. Itis a bird-like airfoil and its polar is available in [14], where it has been computed using XFOIL [15]."

a. It needs to be clarified why the AS6092 airfoil was used in this study, as opposed to other bird-like airfoils available in Ananda and Selig (2018). The authors should include their reasoning for modeling the bird wings with such an airfoil section in the text.

b. Birds' wings are complex lifting surfaces that are characterized by sweep, camber distribution, thickness distribution, twist distribution, and chord distribution [1-5]. Did you consider any of the above wing characteristics in the modeling presented in the paper? Please elaborate in the manuscript text to better describe the modeled bird's wings.

[1] Nachtigall W. and Wieser J. (1966) "Profilmessungen am taubenflúgel", Zeitschriftfur vergleichende Physiologies 52, pp. 333-346.

[2] Oehm H. and Kitzler U. (1975) "On the geometry of the avian wing," Studies on theBiophysics and Physiology of Avian Flight II", NASA TT-F-16901, 1975.

[3] Withers P. C. (1981) "An aerodynamic analysis of bird wings and fixed aerofoils",Journal of Experimental Biology 90, pp. 143-162.

[4] Liu T., Kuykendoll K., Rhew R., and Jones S. (2006) "Avian wing geometry andkinematics", AIAA Journal 44(5), pp. 954-963.

[5] Klän S., Bachmann T., Klaas M., Wagner H., and Schröder W. (2009) "Experimentalanalysis of the flow field over a novel owl based airfoil," Experiments in Fluids 46(5), pp. 975-989.

We agree that our airfoil choice lacked a proper justification and discussion. This point has been discussed in section 1.2 of the present document and led to modifications of the manuscript to account for this remark.

Comment 10: In Section 2.1, line 87, pp. 3: Please indicate how you calculated the wake lambda's wavelength.

We thank the reviewer for pointing this omitted definition. We added the following sentence in the relevant paragraph (which has moved to the start of section 2.1):

Its flapping frequency f is 4 Hz and the wavelength of the wake $\lambda = U/f$ is thus equal to 3.75 m.

Comment 11: Figure 1 – Please indicate in the figure, and also in the text, the size/dimensions of the computational domain (control volume) used to perform the simulations presented in this paper. What are the streamwise/lateral/normal extents?

We added the missing description of the computational domain in the description of the simulated scenarios in section 2.3 of the revised manuscript:

The computational domain used in scenario 1 extends from -3 m to 3 m in the spanwise and vertical directions (which corresponds to 2.2 spans). However, unbounded boundary conditions are applied, so that the flow is not affected by the boundaries of the domain. In the streamwise direction, the inflow is at a distance of 1 m of the bird (0.74 spans), and the outflow is at a distance of 15 m (11.1 spans, or 4 wavelengths). The resolution is of 64 particles per meter, or 86 per span.

Scenario 2 uses a domain with slightly different dimensions as compared to scenario 1. It has the same size in the spanwise direction, extends from -5 to 3 m (-3.7 to 2.2 spans) in the vertical direction, and -2 to 10 m (1.5 to 7.4 spans) in the streamwise direction. Is has a lower resolution than scenario 1, with 27 particles per span.

Comment 12 : In Section 2.1, line 97, pp. 4: "The default values of the kinematic parameters are provided in table 1." – Please include the reference from which the default kinematics of the bird's wing was evaluated.

These kinematic parameters are issued from a previous publication but we omitted to refer to it. We modified the corresponding paragraph to include the reference, and briefly state how the kinematic parameters were determined:

The wing kinematics used in the simulations result from seven joint rotations in the wing skeleton, represented in panel B of Fig. 2. Each joint angle q_i follows a pure sinusoidal trajectory, governed by a unique frequency $f = 4$ Hz, and joint dependent mean value $q_{0,i}$, amplitude A_i and phase ϕ_i. Each joint i thus follows a trajectory defined as $q_i(t) = q_{0,i} + A_i \sin(2\pi f t + \phi_i)$. The parameters $q_{0,i}$, A_i and ϕ_i are adjusted in order to resemble experimentally observed bird wing kinematics. The derivation of the parameters is reported in [16] for the same species, using experimental data from [27]. The resulting values of the kinematic parameters are provided in table 1.

Comment 13: Figure 2 – It would be helpful if the authors would mark the seven joints on the wing.

Figure 2 of the revised paper has been modified and now includes a panel highlighting the seven joint rotations of the wing model. You can see the new figure in Fig. 2 of the present document.

Comment 14: In Section 2.1, Eq. 2, pp. 5: Please include a reference to the computation of the added mass matrix expressed in equation 2.

We thank the reviewer for pointing out this omission. A sentence has been added after the definition of the matrix, giving the corresponding reference:

The matrix $\boldsymbol{M}_a$ of Eq. (2) was obtained using the coefficients provided for an ellipse in Table 4.3 of [24] (using $a = c/2$ and $b = t/2$), and modifying them so that the moment is computed about the quarter of the chord.

Comment 15: The authors used somewhat misleading terminology in the paper to describe their two numerical simulations, namely, experiment 1 and experiment 2. I suggest the authors revise the paper text and use different terminology to describe their numerical simulations, thus avoiding confusion. A better terminology could be 'numerical scenarios' (scenario 1 and scenario 2).

We apologize for the confusion related to our terminology. We agree with the one proposed by the reviewer and made the relevant changes to the manuscript.

Comment 16: Figure 4,5,6 – I suggest the authors normalize the results of the normal force. Maybe, using the bird's weight so that the results could be better interpreted. Also, it could be helpful if the authors would mark the downstroke and upstroke phases of the flapping cycle on the figures.

We thank the reviewer for this suggestion. We divided the values of vertical force by the weight to ease the interpretation of our results.

Comment 17: In Figure 4 – Please elaborate on why the small amplitude 'spikes' appear along the normal force curves (particularly noticeable for the total force). Also, the legend in Figure 4 is missing.

These are due to the reprojection operation that is performed in the fluid solver to ensure the solenoidal property of the vorticity field. This operation occurs every 20 time steps and affects the time-derivative terms in Eq. (4) each time it is performed. We now properly mention this process in the description of the fluid solver:

Finally, particle discretization does not ensure that the vorticity field remains divergence-free. To prevent errors related to this, a reprojection operation is performed on the vorticity field every 20 timesteps. Both of these operations are described in details in [17].

In the revised version of the paper, we modified the figure (now numbered Fig. 7), by interpolating the time derivative at these points from its values just before and after the operation. Since the 'spikes' do not correspond to a physical phenomenon, we consider that this clarifies the figure, without altering the message of the paper.

Comment 18: In Section 4, lines 317-319, pp. 12: "The wake also presents root vortices that have been observedin [18, 6, 7] and spanwise vortices due to time-variations of the wing circulation, as in [4, 9]." – The bird's wakes in Figure 3 seems to be characterized by 2-4 layers of concentrated spanwisevorticity (with alternating sign) aligned at an angle with respect to the horizon. Gurka et al. (2017) referred to the quadruple layers of concentrated spanwise vorticity as 'double branch' features and noticed they exist in the wake of three different bird species when the circulation gradient changes sign, suggesting a common role for unsteady aerodynamics in lift generation for flapping birds. I recommend that the authors refer to Gurka et al. (2017) in their discussion of the simulated bird wake to further augment the manuscript's findings.

We thank the reviewer for the point of comparison suggested. We included the results of Gurka et al. 2017 into our discussion of the wake structure:

In [8], more complex structures are observed. It is a possibility that the multiple layers of vorticity observed in Fig. 5 may correspond to the double branch features reported and discussed in [8], as they also occur in regions of transition between strokes, where the variation of circulation is important. However, our model does not include multiple shedding points from the wing (aside from the shedding of drag-related structures), as would be the case in highly unsteady cases. Thus, the origin of these structures, in our simulations, is in the deformations occurring between the bird and the measurement position.

Comment 19: In Section 4.2, lines 374-375, pp. 13: "Although the quality of the estimation remains acceptable at a distance of seven spans, we see that it degrades as the integration plane moves further downstream from the bird". – Does this mean that the instantaneous lift component can only be recovered using the estimation from near-wake flow measurements behind flapping birds? If so, I think this is a solid finding to be included in the paper.

The component of the instantaneous lift due to the added mass effect can only be estimated by measuring the fluid velocity all around the bird, and the velocities induced by this phenomenon quickly decay as we move away from the wings. Hence, it can not be retrieved from wake measurements. This has been clarified in the conclusions:

We also identify the underlying assumptions of the estimation and quantify them. Added mass is ignored in the estimation, and cannot be retrieved solely from measurements in the bird wake. The only way to properly evaluate its effect is to measure the velocity all around the wings, but we find that its contribution is small in comparison to the circulatory lift.

Concerning the lift due to the wing circulation, its instantaneous evolution can be retrieved from measurements in a single outflow plane. The closer we measure the vorticity field from the wing, the more accurately we can estimate the lift, but we show in our results that even for large distances (7 spans in the non periodic case), we still get an acceptable estimation. This has also been clarified in the conclusions:

The proposed formula can also be used to recover the circulation-related contribution of the instantaneous lift – provided that a time delay is accounted for. The estimated time-dependent lift is found to closely match the reference values at close distances from the bird. While the quality of the estimation degrades, it remains acceptable for streamwise distance of up to seven spans.

Comment 20: In the Conclusions, lines 382-383, pp. 14: "The simulation framework mimics the conditions of a wind tunnel…". – This is somewhat inaccurate since you did not model the wind tunnel walls with an impermeable/no-slip boundary condition in your numerical setup. Therefore, I suggest the authors remove their mention of mimicking wind tunnel tests with their numerical simulations from the paper text.

We agree that this formulation was misleading. We initially meant by this that we subjected the computational domain to a constant inflow velocity equal to the flight velocity of the bird, so that a fixed measurement plane remains at a constant distance of the flying bird, as it would be the case in wind tunnel experiments. Following the suggestion of the reviewer, we removed this sentence from the conclusions.
