# Supplementary material for: Numerical assessment of wake-based estimation of instantaneous lift in flapping flight of large birds
Source: PLoS One. 2023 May 4;18(5):e0284714. doi: 10.1371/journal.pone.0284714 (PMC10159204; doi:10.1371/journal.pone.0284714)
Supplement: Attachment — Submitted filename: Paper_Review_R1.pdf [file pone.0284714.s008.pdf]

Paper Review

Manuscript ID: PONE-D-22-25640R1
Title: Numerical assessment of wake-based estimation of instantaneous lift in flapping flight

The authors have revised the original manuscript thoroughly, adequately answering all of my comments. Nevertheless, I have one last comment on the revised manuscript, which I kindly ask the authors to address:

1. In Section 1.1, lines 164-172, pp. 7: "*The lift contribution of the body-tail ensemble in real birds is still a matter of open discussion. In [21], it is shown that the body-tail of gliding raptors have a higher circulation than the wing at their root, and thus produces more lift. However, in other experimental results comparing the wakes of flapping birds with and without a tail [22], no evidence was found for an aerodynamic effect of the tail. Finally, in [8], the effect of the tail was found to significantly vary with the flight velocity, with higher (resp. lower) lift produced at lower (resp. higher) velocities. Since our scenario concerns steady flapping flight at a high forward velocity, we consider that the assumption of low tail lift is reasonable.*" – The references provided in this paragraph do not support the authors' argument for not including the tail lift in their analysis. First, Usherwood et al. (2020) [Ref. 21] provide details on a similar-size bird (Goshawk) investigated at a Reynolds number (based on the mean chord) of around **124,000**, which is close to the values investigated in this manuscript (180,000). However, in their study, Usherwood et al. (2020) investigated the Goshawk in **gliding** flight mode and not flapping, as studied in this manuscript, thus questioning the applicability of their conclusions to the current manuscript. Secondly, Johansson and Hedenström (2009) [Ref. 22] investigated a small bird in **flapping** flight, the Reynolds numbers studied (based on the mean chord) are **17,500 to 29,500** - an order of magnitude lower than that indicated in the current manuscript (180,000). Thirdly, Henningsson et al. (2010) [Ref. 8] investigated a **gliding** swift at Reynolds numbers (based on the mean chord) in the range of **15,000 to 25,000**, which is not similar to the current study in both the flight mode and Reynolds number regime. Consequently, I suggest the authors supply an alternative argument for not including the tail lift in their computation - one that is more suitable for large flapping birds, such as the one they have studied.
    a. Usherwood, J. R. et al. (2020) 'High aerodynamic lift from the tail reduces drag in gliding raptors', The Journal of Experimental Biology, 223. doi: 10.1242/jeb.214809.
    b. Johansson, L. C. and Hedenström, A. (2009) 'The vortex wake of blackcaps (Sylvia atricapilla L.) measured using high-speed digital particle image velocimetry (DPIV)', The Journal of Experimental Biology, 212(20), pp. 3365–3376. doi: 10.1242/jeb.034454.
    c. Henningsson, P., Muijres, F. T. and Hedenström, A. (2010) 'Time-resolved vortex wake of a common swift flying over a range of flight speeds', Journal of the Royal Society Interface, 8(59), pp. 807–816. doi: 10.1098/rsif.2010.0533.
