# Supplementary material for: Numerical assessment of wake-based estimation of instantaneous lift in flapping flight of large birds
Source: PLoS One. 2023 May 4;18(5):e0284714. doi: 10.1371/journal.pone.0284714 (PMC10159204; doi:10.1371/journal.pone.0284714)
Supplement: Attachment — Submitted filename: response_to_reviewer.pdf [file pone.0284714.s009.pdf]

Numerical assessment of wake-based estimation of instantaneous lift in flapping flight of large birds – second round of review

Victor Colognesi, Renaud Ronsse, Philippe Chatelain
UCLouvain; Institute of Mechanics, Materials and Civil Engineering
Louvain-la-Neuve, Belgium

April 4, 2023

This document replies to the final comment of Reviewer two. It concerns the decision of not including the lift force induced by the tail in our analysis.

Comment: The references provided in [Section 1.1, lines 164-172, pp. 7] do not support the authors' argument for not including the tail lift in their analysis. First, Usherwood et al. (2020) [Ref. 21] provide details on a similar-size bird (Goshawk) investigated at a Reynolds number (based on the mean chord) of around 124,000, which is close to the values investigated in this manuscript (180,000). However, in their study, Usherwood et al. (2020) investigated the Goshawk in gliding flight mode and not flapping, as studied in this manuscript, thus questioning the applicability of their conclusions to the current manuscript. Secondly, Johansson and Hedenström (2009) [Ref. 22] investigated a small bird in flapping flight, the Reynolds numbers studied (based on the mean chord) are 17,500 to 29,500 - an order of magnitude lower than that indicated in the current manuscript (180,000). Thirdly, Henningsson et al. (2010) [Ref. 8] investigated a gliding swift at Reynolds numbers (based on the mean chord) in the range of 15,000 to 25,000, which is not similar to the current study in both the flight mode and Reynolds number regime. Consequently, I suggest the authors supply an alternative argument for not including the tail lift in their computation - one that is more suitable for large flapping birds, such as the one they have studied.

We agree with the reviewer that not modeling the production of lift by the bird tail is a key assumption and requires appropriate arguments. However, in our simulations, the body-tail ensemble does produce lift and drag, through the lifting and dragging line model. The assumption made regarding the tail is that it does not produce more lift than the wings, which can be justified from observations on actual birds.

Regarding the importance of the chord-based Reynolds number, we believe that its impact on the tail lift is not as significant as the strategic choice by the bird to open it or furl it depending on flight regime and wing kinematics. Although the Reynolds number impacts the behavior of the lift coefficient, in fast flight, birds tend to reduce the surface of their tail in order to improve their flight performance, as observed by Thomas [23].

As there is not wind tunnel data about flapping flight of large birds, and thus no data on the actual tail lift of such birds, we need to extrapolate from the existing data collected with other types of birds and flight regimes. We assumed that the role of the tail is governed by flight dynamics, regime and efficiency. Therefore, the references studying fast flapping flight of high aspect ratio migratory birds [22, 8] are preferred compared to the one reporting experiments on raptors in slow, gliding flight [21].

An additional argument for the low aerodynamic lift of the tail can be brought from a recent numerical study from our group analyzing the role of the tail in flight stability and efficiency for large flapping birds [24]. In this study, it has been found that the cost of transport improves significantly for furled tails, especially at high velocities. We can assume that migratory birds thus choose to furl their tails in order to improve their energetic performance in long flights.

The paper has been adapted to include these arguments regarding the choice of not including specifically the tail lift in the computation. Here is the corresponding paragraph (lines 170-175, p. 5 of the revised manuscript), with the added discussion in red:

The lift contribution of the body-tail ensemble in real birds is still a matter of open discussion. In [21] it is shown that the body-tail of gliding raptors have a higher circulation than the wing at their root, and thus produces more lift. However, in other experimental results comparing the wakes of flapping birds with and without a tail [22], no evidence was found for an aerodynamic effect of the tail. Finally, in [8], the effect of the tail was found to significantly vary with the flight velocity, with higher (resp. lower) lift produced at lower (resp. higher) velocities, which is consistent with the observations of [23], who observed that birds tend to furl their tails in fast flight. Finally, it is shown in [24] that the cost of transport of large flapping birds increases with the opening angle of their tails, and that reducing tail lift thus improves flight efficiency. Since our scenario concerns steady flapping flight at a high forward velocity, for a large bird with a high aspect ratio, we consider that the assumption of low tail lift is reasonable.

Two references have been added to the manuscript, and the numbering of the references has been adapted in the revised manuscript. The bibliography below is limited to the references used in the discussion above, and the new references are numbered [23] and [24].

[8] Henningsson P, Mujires FT, Hedenström A. Time-resolved vortex wake of a common swift flying over a range of flight speeds. Journal of The Royal Society Interface. 2010;8(59):807–816.

[21] Usherwood JR, Cheney JA, Song J, Windsor SP, Stevenson JPJ, Dierksheide U, et al. High aerodyamic lift from the tail reduces drag in gliding raptors. Journal of Experimental Biology. 2020;223(3).

[22] Johansson LC, Hedenström A. The vortex wake of blackcaps (*Sylvia atricapilla* L.) measured using high-speed digital particle image velocimetry (DPIV). Journal of Experimental Biology. 2009;212(20):3365–3376.

[23] Thomas ALR On the aerodynamics of birds' tails. Philosophical Transactions of the Royal Society of London. Series B: Biological Sciences. 1993;340(1294):361–380.

[24] Ducci G, Vitucci G, Chatelain P, Ronsse R. On the role of tail in stability and energetic cost of bird flapping flight Scientific Reports. 2022;12(1):22629.
