# Supplementary material for: Numerical assessment of wake-based estimation of instantaneous lift in flapping flight of large birds
Source: PLoS One. 2023 May 4;18(5):e0284714. doi: 10.1371/journal.pone.0284714 (PMC10159204; doi:10.1371/journal.pone.0284714)
Supplement: Attachment — Submitted filename: Review.docx [file pone.0284714.s004.docx]

This work introduces a lift estimation technique for large Reynolds number flapping flight. The paper is well written, and the arguments are convincing. However, there are some major revisions needs to be done to improve the quality of this work:

- This study derives and then validates an instantaneous lift equation using numerical simulation data of a flapping flight model. However, no validation data for the numerical solver had been provided. At the least, velocity profiles and vorticity profiles at the Trefftz planes (using the numerical solver) need to be compared with the experimental data. Although the authors provided a reference for the solver details, this comparison needs to be shown in the main manuscript. Without these comparisons with the experimental data, the validity of the solver remains questionable.
- The authors mentioned that AS6092 (bird-like) airfoil was used for the simulations. However, the simulated bird was *Geronticus Eremita.* This fact largely undermines the validity of this study as a slight difference in camber or curvature (with the actual bird airfoil) would produce significant difference in numerical simulation compared to the experimental data.
- Again, birds’ airfoil can vary significantly along the span in terms of camber and chord length. The authors didn’t mention anything about the differences between the model and the actual bird (*Geronticus Eremita*). A figure showing the wing model as well as the actual bird wing is required to be added in the manuscript.
- Also, this study lacks content. I would expect the authors to include some contents that show variations of lift as a function of the AOA. Since this is a numerical experiment, this can be done after validating the solver data with the level flight of the bird (experimental data).
- It is not clear to me how mathematical operations for the surface integral of the velocity were done to turn it into a surface integral of vorticity*distance (equation 12 in appendix). Can you please show the step by step processes?
- The figures (4a-5d, 7) need line legend.
- I would recommend authors to add more contents/pictures in Figure 3 (evolution of wake vortices). As this is a numerical simulation, the authors should be able to provide a much more detailed picture of evolution of vorticity at the wake.
- To use the lift equation (equation 5), is there any requirement to use threshold on the vorticity maps? Did the authors use any threshold on Figure 3?
